# The Lack of Synapsin Alters Presynaptic Plasticity at Hippocampal Mossy Fibers in Male Mice

**DOI:** 10.1523/ENEURO.0330-23.2024

**Published:** 2024-06-28

**Authors:** Felicitas Bruentgens, Laura Moreno Velasquez, Alexander Stumpf, Daniel Parthier, Jörg Breustedt, Fabio Benfenati, Dragomir Milovanovic, Dietmar Schmitz, Marta Orlando

**Affiliations:** ^1^Charité – Universitätsmedizin Berlin, corporate member of Freie Universität Berlin and Humboldt-Universität zu Berlin, Berlin 10117, Germany; ^2^NeuroCure Cluster of Excellence, Charité – Universitätsmedizin Berlin, corporate member of Freie Universität Berlin and Humboldt-Universität zu Berlin, Berlin 10117, Germany; ^3^Max Delbrück Center for Molecular Medicine in the Helmholtz Association, Berlin 13125, Germany; ^4^Center for Synaptic Neuroscience and Technology, Istituto Italiano di Tecnologia, Genoa 16163, Italy; ^5^IRCCS Ospedale Policlinico San Martino, Genoa 16132, Italy; ^6^German Center for Neurodegenerative Diseases (DZNE) Berlin, Berlin 10117, Germany; ^7^Einstein Center for Neurosciences, Charité – Universitätsmedizin Berlin, corporate member of Freie Universität Berlin and Humboldt-Universität Berlin, Berlin 10117, Germany; ^8^Bernstein Center for Computational Neuroscience, Humboldt-Universität zu Berlin, Berlin 10115, Germany

**Keywords:** hippocampal mossy fibers, presynaptic plasticity, presynaptic potentiation, synapsin, synaptic transmission, synaptic vesicles

## Abstract

Synapsins are highly abundant presynaptic proteins that play a crucial role in neurotransmission and plasticity via the clustering of synaptic vesicles. The synapsin III isoform is usually downregulated after development, but in hippocampal mossy fiber boutons, it persists in adulthood. Mossy fiber boutons express presynaptic forms of short- and long-term plasticity, which are thought to underlie different forms of learning. Previous research on synapsins at this synapse focused on synapsin isoforms I and II. Thus, a complete picture regarding the role of synapsins in mossy fiber plasticity is still missing. Here, we investigated presynaptic plasticity at hippocampal mossy fiber boutons by combining electrophysiological field recordings and transmission electron microscopy in a mouse model lacking all synapsin isoforms. We found decreased short-term plasticity, i.e., decreased facilitation and post-tetanic potentiation, but increased long-term potentiation in male synapsin triple knock-out (KO) mice. At the ultrastructural level, we observed more dispersed vesicles and a higher density of active zones in mossy fiber boutons from KO animals. Our results indicate that all synapsin isoforms are required for fine regulation of short- and long-term presynaptic plasticity at the mossy fiber synapse.

## Significance Statement

Synapsins cluster vesicles at presynaptic terminals and shape presynaptic plasticity at giant hippocampal mossy fiber boutons. Deletion of all synapsin isoforms results in decreased short- but increased long-term plasticity.

## Introduction

Neurotransmission is a fundamental process that enables us to sense the world around us, to react to it, and to think, learn and remember. This process requires high temporal and spatial fidelity, and the energy-expensive and complex regulation of synaptic vesicle trafficking is a prerequisite. A crucial aspect is the spatial arrangement of neurotransmitter-filled vesicles inside the synapse, regulated by the protein family of synapsins (Atias et al., 2019; Sansevrino et al., 2023).

Synapsins are highly abundant phosphoproteins associated with the surface of synaptic vesicles (De Camilli et al., 1990; Cesca et al., 2010), encoded by three mammalian genes (*SYN1*, *SYN2*, *SYN3*; Südhof et al., 1989; Kao et al., 1998). The lack of synapsin I (SynI) and II (SynII) causes vesicle dispersion and shrinks the distal vesicle cluster, the reserve pool (Li et al., 1995; Pieribone et al., 1995; Rosahl et al., 1995). Thus, a major function of synapsins is to control mobilization from the reserve pool, in a phosphorylation-dependent manner (Sihra et al., 1989; Hosaka et al., 1999; Chi et al., 2001). How synapsins preserve this pool is still under debate. Likely mechanisms are: (1) synapsins cross-link the vesicles, acting as tethers (Hirokawa et al., 1989); (2) synapsins form a liquid phase, capturing vesicles in it (Milovanovic et al., 2018; Pechstein et al., 2020); or (3) a mixture of both, since these mechanisms are not mutually exclusive (Zhang and Augustine, 2021; Song and Augustine, 2023; Longfield et al., 2024).

While SynI and SynII are expressed in mature synapses (De Camilli et al., 1983; Browning et al., 1987), synapsin III (SynIII) is primarily expressed during development: after 1 week postnatal its levels decrease drastically (Ferreira et al., 2000) and remain low in adults (Kao et al., 1998). However, in brain regions featuring postnatal neurogenesis, SynIII is still expressed in the adult tissue (Pieribone et al., 2002). This includes the dentate gyrus and hippocampal mossy fibers.

Hippocampal mossy fibers are involved in learning, memory, and spatial navigation (Lassalle et al., 2000; Rolls, 2018). They connect granule cells and CA3 pyramidal cells via mossy fiber boutons, highly plastic synapses (Nicoll and Schmitz, 2005). Activity-dependent changes in neurotransmission can be studied very well in these boutons, because they can react to a wide range of frequencies (Salin et al., 1996) and express presynaptic short- and long-term potentiation (STP, LTP; Zalutsky and Nicoll, 1990; Nicoll and Schmitz, 2005). Recently, a mechanism for short-term memory has been proposed: the formation of a “pool engram”—an increased readily releasable pool (RRP)—which could depend on the vesicle mobilization via synapsins (Vandael et al., 2020). Unlike STP, mossy fiber LTP is still more enigmatic: it is known to be protein kinase A (PKA)-dependent (Weisskopf et al., 1994), but the precise downstream targets and potential parallel mechanisms are not yet clarified (Monday et al., 2018, 2022; Shahoha et al., 2022).

Synapsin-dependent mossy fiber physiology has been investigated in SynI/SynII double knock-out (SynDKO) animals (Spillane et al., 1995; Owe et al., 2009): field recordings revealed impaired frequency facilitation in physiologically relevant ranges (Owe et al., 2009), while LTP was unchanged (Spillane et al., 1995). However, enrichment of SynIII close to the active zone at mossy fiber boutons (Owe et al., 2009) raised the question if the complete knock-out (KO) of synapsins would have further effects on mossy fiber transmission and plasticity.

Here, we examined a glutamatergic synapse that retains SynIII expression in adulthood and asked how neurotransmission and synaptic morphology are changed upon the complete loss of synapsins. We investigated this question in acute slices of SynI/SynII/SynIII triple KO (SynTKO) male mice using a combined approach of transmission electron microscopy (TEM) and electrophysiological field recordings. We observed fewer vesicles in the reserve pool and increased active zone density. Field recordings provided evidence that synapsins are crucial for both STP and LTP in mossy fibers: facilitation and post-tetanic potentiation (PTP) were impaired, while LTP was enhanced.

## Materials and Methods

### Reporting guidelines

This study was reported in accordance with the Sex and Gender Equity in Research (SAGER) guidelines (Heidari et al., 2016) and Animal Research: Reporting of In Vivo Experiments (ARRIVE) guidelines 2.0 (Percie du Sert et al., 2020). The checklist for the SAGER guideline is provided in Table 1, the checklist for the essential 10 of the ARRIVE guideline is provided in Table 2, and the checklist for the recommended set of the ARRIVE guideline is provided in Table 3.

**Table 1.** SAGER guidelines checklist, other studies (applied sciences, cell biology, etc.)

Section/topic	Item number	Checklist item	Reported on page number
General	1	The terms sex/gender used appropriately	Does not apply
Title	2a	Title specifies the sex of animals or any cells, tissues, and other material derived from these	1
	2b	In applied sciences (technology, engineering, etc.), the title indicates if the study model was based on one sex/gender or the application was considered for the use of one specific sex/gender	Does not apply
Abstract	3a	Abstract specifies sex of animals or any cells, tissues, and other material derived from these	4
	3b	In applied sciences (technology, engineering, etc.), the abstract indicates if the study model was based on one sex/gender or the application was considered for the use of one specific sex/gender	Does not apply
Introduction	4a	If relevant, previous studies that show presence or lack of sex or gender differences or similarities are cited	Does not apply
	4b	Mention of whether sex/gender might be an important variant and if differences might be expected	Not performed
Materials and Methods	5a	In cell biological, molecular biological, or biochemical experiments, the origin and sex chromosome constitutions of cells or tissue cultures are stated. If unknown, the reasons are stated	Does not apply
	5b	For studies testing devices or technology, explanation of whether the product will be applied or used by all genders and if it has been tested with a user's gender in mind	Does not apply
	5c	If relevant, description of how sex/gender was considered in the design	Does not apply
	5d	For in vivo and in vitro studies using primary cul­tures of cells, or cell lines from humans or animals, or ex vivo studies with tissues from humans or animals, the sex of the subjects or source donors is stated (except for immortalized cell lines, which are highly transformed)	7
Results	6	For studies using animal models, present a sex breakdown of the animals^a^	Does not apply
Discussion	7	If relevant, potential implications of sex/gender on the study results and analyses, including the extent to which the ﬁndings can be generalized to all sexes/genders in a population	28

Adapted from Heidari et al. (2016).

a These points extend beyond the original SAGER table.

**Table 2.** The ARRIVE guidelines 2.0 checklist: the essential 10

Item	Item number		Recommendation	Reported in section
Study design	1	a.	For each experiment, provide brief details of study design including: the groups being compared, including control groups. If no control group has been used, the rationale should be stated	Materials and Methods, Study design and Results
		b.	The experimental unit (e.g., a single animal, litter, or cage of animals)	Materials and Methods, Study design and Table 5
Sample size	2	a.	Specify the exact number of experimental units allocated to each group and the total number in each experiment. Also indicate the total number of animals used	Materials and Methods, Study design and Table 5
		b.	Explain how the sample size was decided. Provide details of any a priori sample size calculation, if done	Materials and Methods, Study design
Inclusion and exclusion criteria	3	a.	Describe any criteria used for including and excluding animals (or experimental units) during the experiment and data points during the analysis. Specify if these criteria were established a priori. If no criteria were set, state this explicitly	Materials and Methods, Field recordings
		b.	For each experimental group, report any animals, experimental units, or data points not included in the analysis and explain why. If there were no exclusions, state so	Materials and Methods, Tables 5 and 6
		c.	For each analysis, report the exact value of n in each experimental group.	Materials and Methods, Study design and Table 5
Randomi­zation	4	a.	State whether randomization was used to allocate experimental units to control and treatment groups. If done, provide the method used to generate the randomization sequence	Materials and Methods, Study design
		b.	Describe the strategy used to minimize potential confounders such as the order of treatments and measurements or animal/cage location. If confounders were not controlled, state this explicitly	Materials and Methods, Study design
Blinding	5		Describe who was aware of the group allocation at the different stages of the experiment (during the allocation, the conduct of the experiment, the outcome assessment, and the data analysis)	Materials and Methods, Study design
Outcome measures	6	a.	Clearly define all outcome measures assessed (e.g., cell death, molecular markers, or behavioral changes)	Results; Materials and Methods, TEM and Table 5
		b.	For hypothesis-testing studies, specify the primary outcome measure, i.e., the outcome measure that was used to determine the sample size	Does not apply
Statistical methods	7	a.	Provide details of the statistical methods used for each analysis, including the software used	Materials and Methods, Statistics
		b.	Describe any methods used to assess whether the data met the assumptions of the statistical approach and what was done if the assumptions were not met	Materials and Methods, Statistics
Experimental animals	8	a.	Provide species-appropriate details of the animals used, including species, strain and substrain, sex, age or developmental stage, and, if relevant, weight	Materials and Methods, Study design and key resources table
		b.	Provide further relevant information on the provenance of animals, health/immune status, genetic modification status, genotype, and any previous procedures	Materials and Methods, Study design; Results; Discussion
Experimental procedures	9	a.	For each experimental group, including controls, describe the procedures in enough detail to allow others to replicate them, including: what was done, how it was done, and what was used	Materials and Methods
		b.	When and how often	Materials and Methods, TEM, Table 5
		c.	Where (including detail of any acclimatization periods)	Materials and Methods, Acute slice preparation, Field re­cordings, and TEM
		d.	Why (provide rationale for procedures)	Introduction; Results; Discussion; Materials and Methods
Results	10	a.	For each experiment conducted, including independent replications, report the summary/descriptive statistics for each experimental group, with a measure of varia­bility where applicable (e.g., mean and SD or median and range)	Results, text and figure legends
		b.	If applicable, the effect size with a confidence interval	Does not apply

Adapted from Percie du Sert et al. (2020).

**Table 3.** The ARRIVE guidelines 2.0: the recommended set

Item	Item number		Recommendation	Reported in section
Abstract	11		Provide an accurate summary of the research objectives, animal species, strain and sex, key methods, principal findings, and study conclusions	Abstract
Background	12	a.	Include sufficient scientific background to understand the rationale and context for the study, and explain the experimental approach	Introduction; Results; Discussion
		b.	Explain how the animal species and model used address the scientific objectives and, where appropriate, the relevance to human biology	Introduction; Results; Discussion
Objectives	13		Clearly describe the research question, research objectives, and, where appropriate, specific hypo­theses being tested	Abstract; Introduction; Results; Discussion
Ethical statement	14		Provide the name of the ethical review committee or equivalent that has approved the use of animals in this study and any relevant license or protocol numbers (if applicable). If ethical approval was not sought or granted, provide a justification	Materials and Methods, Ethics statement
Housing and husbandry	15		Provide details of housing and husbandry condi­tions, including any environmental enrichment	Materials and Methods, Acute slice preparation
Animal care and monitoring	16	a.	Describe any interventions or steps taken in the experimental protocols to reduce pain, suffering, and distress	Materials and Methods, Acute slice preparation
		b.	Report any expected or unexpected adverse events	Does not apply
		c.	Describe the humane endpoints established for the study, the signs that were monitored, and the frequency of monitoring. If the study did not have humane endpoints, state this	Not performed
Interpretation/scientific implications	17	a.	Interpret the results, taking into account the study objectives and hypotheses, current theory, and other relevant studies in the literature	Discussion
		b.	Comment on the study limitations including potential sources of bias, limitations of the animal model, and imprecision associated with the results	Discussion
Generalizability/translation	18		Comment on whether, and how, the findings of this study are likely to generalize to other species or experimental conditions, including any relevance to human biology (where appropriate)	Discussion
Protocol registration	19		Provide a statement indicating whether a protocol (including the research question, key design features, and analysis plan) was prepared before the study and if and where this protocol was registered	Not performed
Data access	20		Provide a statement describing if and where study data are available	Data Availability
Declaration of interests	21	a.	Declare any potential conflicts of interest, including financial and non-financial. If none exist, this should be stated	Conflict of interest statement
		b.	List all funding sources (including grant identifier) and the role of the funder(s) in the design, analysis, and reporting of the study	Acknowledgments

Adapted from Percie du Sert et al. (2020).

### Ethics statement

All animal experiments were carried out according to the guidelines stated in Directive 2010/63/EU of the European Parliament on the protection of animals used for scientific purposes and were approved by the animal welfare committee of Charité – Universitätsmedizin Berlin and the Landesamt für Gesundheit und Soziales Berlin (permit T 0100/03 and permit G 0146/20).

### Study design

In this study, only male mice were used for experiments to exclude possible indirect estrogen effects on mossy fiber plasticity (Harte-Hargrove et al., 2013). In electrophysiological recordings, C57BL/6J control mice [research resource identifier (RRID):IMSR_JAX:000664] were compared with SynTKO mice (RRID:MMRRC_041434-JAX) in two age groups: one younger group (4–6 weeks of age), which is referred to as presymptomatic, and one older group (17–19 weeks of age), which is referred to as symptomatic. These terms describe the phenotype before and after the onset of epileptic seizures in SynTKO animals, respectively (Farisello et al., 2013). SynTKO mice were purchased from the Jackson Laboratory (RRID:SCR_004633) and were based on the work of Gitler and coworkers (Gitler et al., 2004). The presymptomatic SynTKO data were obtained from two different cohorts. We received the first cohort from Prof. Dr. Fabio Benfenati (Instituto Italiano di Tecnologia). The second cohort from Dr. Dragomir Milovanovic (DZNE) was housed and bred in the Charité animal facility (Forschungseinrichtungen für Experimentelle Medizin). Symptomatic SynTKO animals and all control animals were also bred and born in the Charité animal facility. For each experiment, we were aiming for at least three biological replicates (animals) per group. Depending on experimental success (how many recordings needed to be excluded, technical failures), we added more animals per group.

#### Field recordings

Data from both presymptomatic SynTKO cohorts were pooled, because they were not significantly different (Table 4). Field recording experiments in all four groups [wild-type (WT), SynTKO, presymptomatic, symptomatic] were repeated with at least three mice from more than one liter (Table 5). Variable *s* represents the number of recorded slices, while *a* reports the number of animals. We were not blinded toward the genotype, because the phenotype was too strong.

**Table 4.** Statistical comparison for experimental values between two cohorts of presymptomatic SynTKO animals

Experiment	Measure	Presynaptic SynTKO animals from Italy	Presynaptic SynTKO animals from Berlin
Input–output	Slope of simple linear regression	1.341	0.833
	Ranges of 95% confidence band	0.5494–2.134	0.09287–1.573
	*p* value slopes (ANCOVA)	0.69	
1 Hz facilitation	Median	4.440	5.955
	Interquartile range	3.760–6.260	3.753–11.41
	*p* value (Mann–Whitney U test)	0.3473	
PPR	Median	3.557	4.545
	Interquartile ranges	2.299–4.260	2.568–6.035
	*p* value (Mann–Whitney U test)	0.0899	
PTP (norm. fEPSP)	Median	3.469	4.116
	Interquartile ranges	2.118–4.589	3.374–7.144
	*p* value (Mann–Whitney U test)	0.1151	
LTP after 30 min (norm. fEPSP)	Median	245.2	228.8
	Interquartile ranges	198.3–302.9	182.5–384.4
	*p* value (Mann–Whitney U test)	0.8793	

**Table 5.** Overview of slice and animal numbers for different experimental groups for field recordings

*s* = number of slices *a* = number of animals	C57BL/6J (4–6 weeks)	Synapsin TKO (4–6 weeks)		C57BL/6J (17–19 weeks)	Synapsin TKO (18–19 weeks)
		From Italy (4–5 weeks)	From Berlin (4–6 weeks)		
Recorded	*s* = 67	*s* = 39	*s* = 18	*s* = 29	*s* = 24
	*a* = 12	*a* = 9	*a* = 7	*a* = 5	*a* = 5
Included	*s* = 31	*s* = 24	*s* = 14	*s* = 17	*s* = 19
	*a* = 12	*a* = 9	*a* = 4	*a* = 4	*a* = 5
Input–output ratio	*s* = 31	*s* = 23	*s* = 14	*s* = 17	*s* = 18
	*a* = 12	*a* = 9	*a* = 4	*a* = 4	*a* = 5
PPR	*s* = 31	*s* = 20	*s* = 14	*s* = 17	*s* = 19
	*a* = 12	*a* = 8	*a* = 4	*a* = 4	*a* = 5
1 Hz facilitation	*s* = 31	*s* = 15	*s* = 12	*s* = 17	*s* = 19
	*a* = 12	*a* = 5	*a* = 4	*a* = 4	*a* = 5
25 Hz stimulation	*s* = 11	*s* = 4	*s* = 8		
	*a* = 5	*a* = 3	*a* = 3		
PTP + LTP	*s* = 15	*s* = 13	*s* = 10		
	*a* = 6	*a* = 6	*a* = 3		

Note: all numbers reported for individual experiments are only from the included subset of recordings.

Recordings were excluded when they had a baseline field excitatory postsynaptic potential (fEPSP) smaller than two times noise (Table 6). Noise was ∼25 µV, so the baseline fEPSP amplitude needed to be at least 50 µV to be included. Furthermore, to include only mossy fiber-specific recordings, we applied 1 µM (2*S*,1'*R*,2'*R*,3'*R*)-2-(2,3-dicarboxycyclopropyl)glycine (DCG-IV; 0975, Tocris Bioscience) at the end of each experiment (Kamiya et al., 1996). If the suppression was 75% or more, the recording was included (Table 6). We were not able to measure input–output curves for all animals. For those cases where it was not recorded with different input strengths, we plotted only one value based on the averaged baseline values for presynaptic fiber volley (PFV) and fEPSP, respectively. If the PFV could not be measured unambiguously, this measurement was excluded from the input–output graph. If the 1 or 25 Hz induction failed, the respective measurements were excluded from analysis, but all other parameters from the same experiment were included. The same was true for some recordings, in which no 25 Hz stimulation and thus no PTP and LTP recordings were conducted. Due to the pooled data from the two presymptomatic SynTKO cohorts, there is an imbalance between numbers from WT and numbers from SynTKO animals in high-frequency recordings. If possible, two mice with different genetic backgrounds were recorded on the same day to minimize variability due to experimental day.

**Table 6.** Exclusion reasons for field recordings

Excluded recordings	C57BL/6J (4–6 weeks)	Synapsin TKO (4–6 weeks)	C57BL/6J (17–19 weeks)	Synapsin TKO (18–19 weeks)
Baseline fEPSP <50 µV	4	2	2	2
DCG-IV effect <75%	27	14	11	4
Other reasons	6	2	0	1
Total number	36	19	12	5

Note that several reasons can apply to the same recording.

#### Transmission electron microscopy

For ultrastructural investigation of mossy fiber boutons, we analyzed mossy fiber boutons from three WT and three SynTKO male mice aged 4–6 weeks. Mice from the two presymptomatic SynTKO cohorts were pooled. We imaged serial sections from 18 WT and 16 SynTKO mossy fiber boutons, respectively. We measured bouton complexity, vesicle number, and mean nearest neighbor distance (MNND) between vesicles in 2D images. Measures of active zone density, active zone area, and docked synaptic vesicle density were obtained by manually annotating partial 3D reconstructions of mossy fiber boutons (total volume of presynaptic boutons analyzed, 48.2 µm^3^; average volume of each fraction of presynaptic bouton, 0.72 ± 0.28 µm^3^; data not shown). Slices from each animal were either treated with forskolin (FSK) or allocated as the control. Allocation of slices to the treatment or control group was block-randomized. Replicates of 17 (WT), 16 (WT + FSK), 16 (SynTKO), and 18 (SynTKO + FSK) mossy fiber boutons were analyzed. Number *n* represents the number of partial presynaptic bouton reconstructions. The experimenter was blinded to the treatment of slices from fixation of the slices until the end of analysis. Due to the strong reduction in vesicle density of SynTKO synapses, blinding during analysis was only possible between treatment groups, but not between genotypes.

### Acute slice preparation

Animals were kept in a 12/12 h light/dark cycle, and water and food were provided *ad libitum*. Cages offered shelter in the form of a house and tubes. Cages of SynTKO animals were kept in remote shelves to minimize exposure to light and possible noises. The first cohort of presymptomatic SynTKO animals was imported from Italy and allowed to sit in the Charité animal facility for several days before the experiments started. After the transfer from the animal facility to the preparation room, all animals were allowed to acclimate to the new surroundings for at least half an hour. Acute brain slices were prepared as follows: mice were anesthetized under the hood with isoflurane and quickly killed with sharp scissors. The brain was taken out and placed in oxygenated ice-cold sucrose–artificial cerebrospinal fluid (S-ACSF) for 3 min to allow equilibration. S-ACSF contained the following (in mM): 50 NaCl, 25 NaHCO_3_, 10 glucose, 150 sucrose, 2.5 KCl, 1 NaH_2_PO_4_, 0.5 CaCl_2_, 7 MgCl_2_. All solutions were saturated with 95% O_2_ (*v*/*v*)/5% CO_2_ (*v*/*v*) and had a pH of 7.4 and an osmolarity of 340 mOsm. Hemispheres were separated, and 300 µm/150 µm (field recordings/electron microscopy) thick sagittal sections were cut from both hemispheres with a vibratome [VT1200 S, Leica Biosystems (RRID:SCR_018453)]. Slices were stored in a submerged chamber in oxygenated S-ACSF at 34°C for half an hour before they were moved to another submerged chamber with ACSF at room temperature. There, slices were kept until the start of experiments. ACSF had an osmolarity of 300 mOsm and a pH of 7.4 and contained (in mM): 119 NaCl, 26 NaHCO_3_, 10 glucose, 2.5 KCl, 1 NaH_2_PO_4_, 2.5 CaCl_2_, 1.3 MgCl_2_. All chemicals were purchased from Sigma-Aldrich.

### Field recordings

Slices were kept in a submerged chamber with ACSF at least 30 min and up to 8 h before the start of recordings. Slices were placed in a recording chamber under a microscope and were continuously superfused with oxygenated ACSF at room temperature at a rate of ∼2.5 ml/min. The recording electrode was fixed in a headstage of the amplification system [Axon Instruments, MultiClamp 700A/700B (RRID:SCR_018455)]. Stimulation and recording electrode units were placed on micromanipulators (Mini 23/25, Luigs & Neumann) for a precise movement control via a control system (SM-5/-7/-10, Luigs & Neumann).

The stimulation and recording electrodes were prepared from silver wires (AG-8W and E-205, Science Products). Glass pipettes were made from borosilicate capillaries (GB150EFT-10, Science Products or 1403005, Hilgenberg) with a pipette puller (PC-10, Narishige or DMZ-Universal Puller, Zeitz-Instrumente) and were broken at the tip with a micro forge (MF-830, Narishige) to receive low-resistance pipettes. Electrodes were placed in the hilus of the dentate gyrus near the granule cell layer (stimulation) and within the *stratum lucidum* of the area CA3 of the hippocampus (recording), respectively.

Stimulations were executed with a stimulation box [ISO-Flex, AMPI (RRID:SCR_018945)], and stimulation patterns were controlled with a Master-8 generator [AMPI (RRID:SCR_018889)]. Igor Pro [version 6, WaveMetrics (RRID:SCR_000325)] was used for signal acquisition. The Axon MultiClamp amplifier [700A/700B, Molecular Devices (RRID:SCR_018455)] was used in the current clamp mode *I* = 0, with filtering of 2 kHz. Signals were digitized (Axon Digidata 1550B, Molecular Devices/BNC-2090; National Instruments Germany) at a rate of 20 kHz. Mossy fiber signals were searched by placing the stimulation and recording electrodes at different locations in the hilus and stratum lucidum, respectively. Once a mossy fiber input was obtained, the recording was started, and the mossy fibers were stimulated at 0.05 Hz.

The standard stimulation frequency was 0.05 Hz throughout the experiment, unless otherwise stated. The recorded sweep length was 0.5 s except for the high-frequency stimulation at 25 Hz where 5.5 s were recorded. First, input–output relations were recorded by applying different input currents via the stimulation box. The strength of the input current was adjusted to yield a specific PFV size: 0.05 mV, 0.1 mV, 0.2 mV, 0.3 mV and maximum (maximal stimulation strength of 10 mA). Each input strength was recorded for three sweeps. Afterward, a medium stimulation strength was chosen, and a baseline was recorded for at least 10 sweeps. Then, the stimulation frequency was increased to 1 Hz for 20 sweeps for recording of frequency facilitation. Afterwards, when fEPSP amplitudes declined to baseline level again, a paired-pulse with an interstimulus interval of 50 ms was applied for three sweeps. Then, a baseline was recorded for 10 min (30 sweeps, except for once when only 20 sweeps were recorded) before a high-frequency train of stimuli was given: four times 125 pulses at 25 Hz every 20 s with a recorded sweep length of 5.5 s. PTP and subsequently LTP were measured for at least 30 min after the tetanus. Mossy fiber purity of signals was verified at the end of each recording with the application of 1 µM DCG-IV (0975, Tocris Bioscience). All recordings with a suppression of at least 75% of the signal were used for analysis.

### Field recording analysis

Field recordings were analyzed with Igor Pro [versions 6 and 8, WaveMetrics (RRID:SCR_000325)] and the installed plugin NeuroMatic (RRID:SCR_004186) as well as Microsoft Excel (RRID:SCR_016137). Igor Pro is commercially available at https://www.wavemetrics.com/products/igorpro, and Microsoft Excel is commercially available at https://www.microsoft.com/de-de/microsoft-365/excel. PFVs were measured from peak to peak. fEPSP amplitudes were baseline-corrected and measured ±2 ms around the peak. For input–output curves, the mean value of the three sweeps at the same stimulation strength was taken, except for the cases in which no input–output curve was recorded: here, we took the average size of PFV and fEPSP amplitudes from the initial baseline. fEPSP amplitudes during 1 Hz facilitation were normalized to the initial baseline (10 sweeps, 3 min). The paired-pulse ratio (PPR) was calculated as the ratio between the second to the first fEPSP amplitude. The stated PPR refers to the first of three paired stimulations. For analysis of the high-frequency trains, we normalized the fEPSP amplitudes to the baseline before (30 sweeps, 10 min). We also evaluated the PFV size for a subset of fEPSPs of the 25 Hz trains. We measured the PFV for stimuli 10–15 and averaged those six values for the first and fourth stimulation train, respectively (Fig. 2*f*). Also, we calculated the ratio of those averaged values between fourth and first stimulation train, to compare the relative loss of PFV size (Fig. 2*g*). Values for PTP and LTP were normalized to the average of the recorded baseline before high-frequency stimulation (30 sweeps, 10 min). Values for LTP were the averaged fEPSP amplitudes from minute 20–30 (30 sweeps) after induction. At the end of the recording, specificity was verified by application of DCG-IV. We averaged the last 15 sweeps of DCG-IV wash-in for quantification. Recordings, in which the suppression was <75% were not counted as mossy fiber-specific and were not included in the analysis.

### Transmission electron microscopy

After preparation, acute slices were allowed to recover in ACSF at room temperature for at least 30 min. Subsequently, we induced chemical LTP in half of the slices by incubating them in 50 µM forskolin (FSK; AG-CN2-0089-M050, Cayman Chemical), dissolved in DMSO, for 15 min at room temperature in oxygenated ACSF (Orlando et al., 2021). The other half of the slices (controls) were incubated in ACSF containing the same concentration of DMSO as the treatment group. Treatment was allocated following a block randomization design. Subsequently, we moved the slices under a chemical hood where fixation, postfixation, staining, dehydratation, and infiltration steps were performed. We fixed proteins by immersing brain slices in a solution containing 1.25% glutaraldehyde (E16216, Science Services) in 66 mM NaCacodylate (E12300, Science Services) buffer for 1 h at room temperature. After extensive washing in 0.1 M NaCacodylate, buffer slices were postfixed in 1% OsO_4_ in 0.1 M NaCacodylate buffer for 1 h at room temperature. Slices were then washed and stained en bloc with 1% uranyl acetate (1.08473, Merck Millipore) in dH_2_O and dehydrated in solutions with increasing ethanol concentration (70, 80, 96, and 100%). Final dehydration was obtained by incubating slices in propylene oxide [20401, Electron Microscopy Sciences (EMS)]. The infiltration of epoxy resin was obtained by serial incubations in increasing resin/propylene oxide dilutions (1:3; 1:1; 3:1). Samples were finally flat embedded in Epon (E14120-DMP, Science Services) for 48 h at 60°C. The *stratum lucidum* in the CA3 region of the hippocampus was identified in 700nm semithin sections stained with Toluidine blue (Sigma-Aldrich) using a light microscope (Olympus); 70 nm serial sections of these regions of interest were cut with an Ultracut UCT ultramicrotome (Leica Microsystems) equipped with an Ultra 45° diamond knife (DiATOME) and collected on pioloform-coated copper slot grids (EMS2010-Cu, Science Services). If not otherwise stated, all chemicals were purchased from EMS and sold by Science Services.

### Electron microscopy imaging of serial sections and 3D reconstructions

A magnification of 7 kx was used to determine the localization of mossy fiber boutons. Subsequently, synapses were imaged at 20 kx (pixel size 2.2 nm) using an EM 900 TEM (Carl Zeiss, RRID:SCR_021364) operated at 80 keV and equipped with a Proscan 2K Slow-Scan CCD-Camera (Carl Zeiss). The *stratum lucidum* of the hippocampal region CA3 was easily distinguishable for the presence of big mossy fiber boutons and for its localization just above the pyramidal cell layer. Mossy fiber boutons were recognized by their large size, their position close to CA3 pyramidal cell bodies, and a large number of presynaptic vesicles (Rollenhagen et al., 2007), including large clear and large dense-core vesicles. Serial images of individual mossy fiber boutons were manually acquired in manually collected serial sections using the ImageSP software (TRS & SysProg) and aligned using the Midas script of the IMOD software (RRID:SCR_003297). The ImageSP software is commercially available at https://sys-prog.com/en/software-for-science/imagesp/, and the IMOD software is freely available at https://bio3d.colorado.edu/imod/. Synaptic profiles were manually segmented in each image of series belonging to the same mossy fiber bouton. Active zones were identified as the area of the membrane opposite to a postsynaptic density/spine profile and with vesicles attached and accumulated next to the presynaptic membrane.

### Active zone analysis

When active zone profiles were visible in at least two sequential serial images, they were traced with the IMOD open line tool and rendered in a 3D model as meshed surfaces. With this criterion we excluded active zones that were only visible in one serial section but included some partially reconstructed active zones that were found at the border of the bouton volume. It is important to note that our partial 3D reconstructions are limited to a fraction of the mossy fiber bouton, measuring, on average, 0.72 ± 0.28 µm^3^ (data not shown). To account for this limitation, the active zone density values were obtained by normalizing the number of 3D reconstructed active zones to the volume of the presynaptic bouton in the partial reconstruction.

### Synaptic vesicle analysis

To analyze synaptic vesicles, we used a machine learning-based algorithm that we had previously developed (Imbrosci et al., 2022). It is freely available at https://github.com/Imbrosci/synaptic-vesicles-detection. Briefly, we manually traced the contour of the mossy fiber bouton using Fiji (RRID:SCR_002285), freely available at https://fiji.sc/. This approach allowed us to obtain a measure of the bouton area and to create a mask over parts of the image which were not relevant for analysis. Synaptic vesicle analysis was performed automatically in a batch. From all images, the number of vesicles and the MNND were obtained. A tutorial with a demonstration of the tool can be found online at https://www.youtube.com/watch?v=cvqIcFldVPw. A limitation of this approach is that the algorithm detects vesicles attached to the plasma membrane with a lower accuracy compared with how it detects isolated vesicles. To overcome this limitation, we performed a manual analysis of docked vesicles at each 3D reconstructed active zone. The docked vesicle density values were obtained by normalizing the number of docked vesicles to the volume of the presynaptic bouton in the partial reconstruction.

### Statistics

For statistical analysis, we used the GraphPad Prism software [GraphPad Prism version 8.4.0 for Windows (RRID:SCR_002798)] and R Project for Statistical Computing (version 4.2.2, RRID:SCR_001905) in RStudio (version 2022.12.0, RRID:SCR_000432). GraphPad Prism is commercially available at https://www.graphpad.com/, and R Project for Statistical Computing is freely available at https://www.r-project.org/. Distribution of data residuals was visually inspected using a *Q*–*Q* plot and/or tested for normality (D’Agostino and Pearson’s test) before evaluating them statistically, to understand if the distribution was Gaussian or non-Gaussian. Individual data points are shown as median ± quartiles, mean values with borders of 95% confidence intervals, or mean ± SEM. The alpha level for statistical significance was 0.05.

#### GraphPad Prism

**Figure 1.**
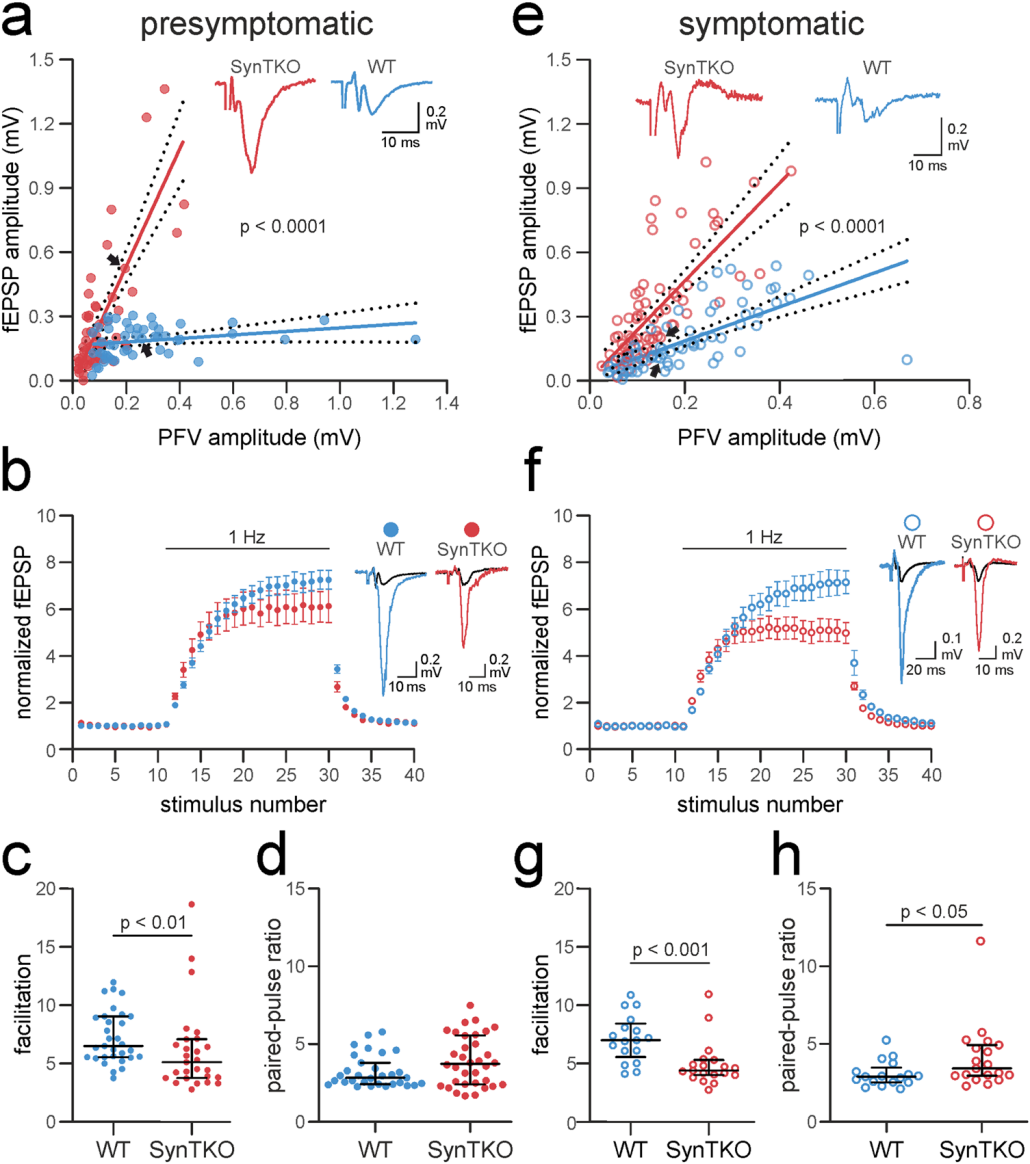
Increased excitability, but reduced facilitation, at mossy fibers of presymptomatic and symptomatic SynTKO mice. ***a***, Excitability was increased in brain slices from presymptomatic SynTKO mice (red, 37 slices from 13 animals) compared with WT mice (blue, 31 slices from 12 animals). Pooled fEPSP amplitudes (mV) were plotted against pooled PFV amplitudes (mV) and fitted with a simple linear regression. The slopes of the linear regressions were significantly different (*p* < 0.0001, tested with a two-tailed ANCOVA). The 95% confident bands are shown as dotted lines around the fit. The black arrows indicate the data points corresponding to the example traces shown in the inset. Inset, Example traces from WT (blue) and SynTKO (red) slices with similar PFV amplitudes. Note the difference in the corresponding fEPSP amplitude. ***b***, ***c***, Frequency facilitation is reduced in presymptomatic SynTKO (red) compared with WT (blue) slices. ***b***, Averaged normalized fEPSP amplitudes ± SEM from all WT (blue, 31 slices from 12 animals) and SynTKO (red, 27 slices from 9 animals) recordings plotted against the number of stimuli. Stimuli 1–10 were given with a frequency of 0.05  Hz, Stimuli 11–30 with 1  Hz, and Stimuli 31–41 with a frequency of 0.05  Hz again. Both time and the interaction between genotype and time were significantly different in a mixed-effect model (*p* < 0.0001; *p* < 0.001). Post hoc Sidak’s test for multiple comparisons revealed no significant differences. Right, Example fEPSP amplitudes from WT (blue) and SynTKO (red) recordings at the 20th 1  Hz stimulus. Respective baseline fEPSP amplitudes are shown in black. ***c***, fEPSP amplitudes at the 20th stimulus at 1  Hz for individual WT (blue dots; 31 slices from 12 animals) and SynTKO (red dots; 27 slices from 9 animals) recordings. Median values ± interquartile ranges are shown in black. Facilitation was significantly different (*p* = 0.0089; Mann–Whitney *U* test). ***d***, PPR for presymptomatic SynTKO and age-matched control animals. Dots represent PPR from individual recordings from WT (blue dots, 31 slices from 12 animals) and SynTKO (red dots, 34 slices from 12 animals) slices, calculated as the ratio of second to first fEPSP amplitude. The interstimulus interval was 50  ms. Median values ± interquartile ranges are depicted in black. Ranks were compared in a Mann–Whitney U test and were not significantly different (*p* = 0.133). ***e***, Excitability was increased in recordings from symptomatic SynTKO mice (red; 18 slices from 5 animals) compared with WT mice (blue; 17 slices from 4 animals). Pooled fEPSP amplitudes (mV) were plotted against pooled PFV amplitudes (mV) and fitted with a simple linear regression. The slopes of the linear regressions were significantly different (*p* < 0.0001, tested with a two-tailed ANCOVA). The 95% confidence bands are shown as dotted lines around the fit. Inset**,** Example traces from WT (blue) and SynTKO (red) animals with similar PFV amplitudes. Note the difference in the corresponding fEPSP amplitude. ***f***, ***g***, Frequency facilitation was reduced in symptomatic SynTKO (red) compared with WT (blue) animals**. *f***, Averaged normalized fEPSP amplitudes ± SEM from all WT (blue; 17 slices from 4 animals) and SynTKO (red; 19 slices from 5 animals) recordings plotted against the number of stimuli. Stimuli 1–10 were given with a frequency of 0.05  Hz, Stimuli 11–30 with 1  Hz, and Stimuli 31–41 with a frequency of 0.05  Hz again. Both time and the interaction between genotype and time were significantly different in a mixed-effect model (*p* < 0.0001). Post hoc Sidak’s test for multiple comparisons revealed significant differences (*p* < 0.05) for two time points. Right, Example fEPSP amplitudes from WT (blue) and SynTKO (red) animals at the 20th 1  Hz stimulus. Respective baseline fEPSP amplitudes are shown in gray. ***g***, fEPSP amplitudes at the 20th stimulus at 1  Hz for individual WT (blue dots, 17 slices from 4 animals) and SynTKO (red dots, 19 slices from 5 animals) recordings. Median values ± interquartile ranges are shown in black. Facilitation was significantly different (*p* = 0.0009; tested with Mann–Whitney *U* test). ***h***, PPR for symptomatic SynTKO and age-matched control animals. Top, Example traces for a paired-pulse from WT (dark blue) and SynTKO (dark red) recordings, respectively. Bottom, Dots represent PPR from individual recordings from WT (dark blue dots, 17 slices from 4 animals) and SynTKO (dark red dots, 19 slices from 5 animals) slices, calculated as the ratio of second to first fEPSP amplitude. The interstimulus interval was 50  ms. Median values ± interquartile ranges are depicted in black. Ranks were compared in a Mann–Whitney U test and were significantly different (*p* = 0.0325).

**Figure 2.**
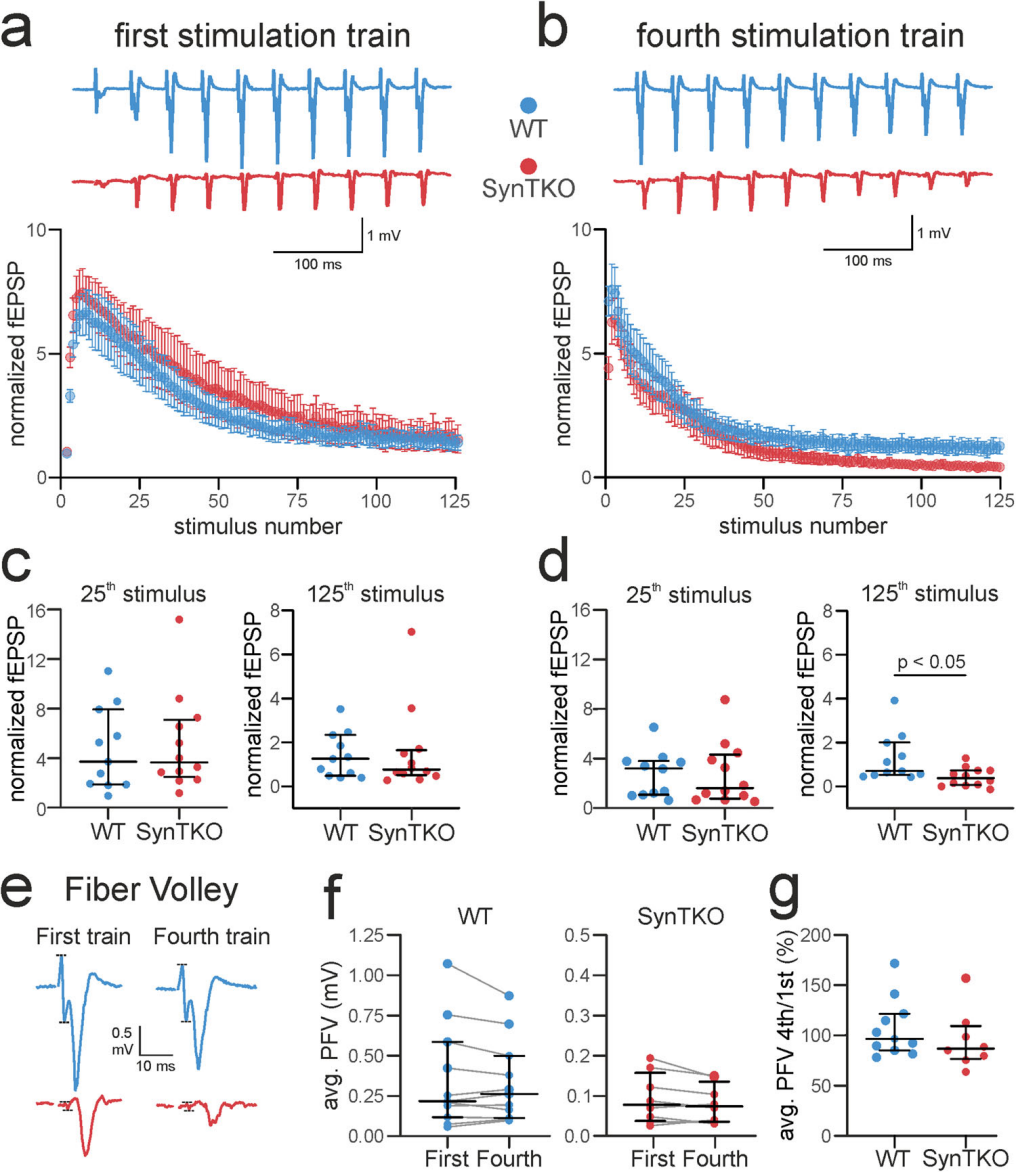
Faster depression during high-frequency stimulation in SynTKO mice. High-frequency stimulation comprised four trains of 125 pulses at 25  Hz with an interval of 20  s between the first stimuli of consecutive trains. ***a***, Top, Example traces show fEPSP amplitudes of mossy fibers from WT (blue) and SynTKO (red) slices in response to the first 10 stimuli of the first high-frequency stimulation train. Bottom, Normalized averaged fEPSP amplitudes plotted against the number of stimuli of the first high-frequency stimulation train for WT (blue, 11 slices from 5 animals) and SynTKO (red, 12 slices from 6 animals) recordings. A mixed-effect model revealed no significant difference between genotypes (*p* = 0.59), but significant differences (*p* < 0.0001) for the factor time (stimulus). A post hoc Sidak’s test for multiple comparisons revealed no significant differences for single time points. ***b***, Top**,** Example traces show fEPSP amplitudes of WT (blue) and SynTKO (red) animals in response to the first 10 stimuli of the fourth high-frequency stimulation train. Bottom, Normalized averaged fEPSP amplitudes plotted against number of stimuli of the first high-frequency stimulation train for WT (blue, 11 slices from 5 animals) and SynTKO (red, 12 slices from 6 animals) animals. The factor time (stimulus) was significantly different in a mixed-effect model (*p* < 0.0001), while the genotype and the interaction of both were not (*p* = 0.22 and *p* > 0.99). A post hoc Sidak’s test for multiple comparisons revealed no significant differences for single time points. ***c***, Normalized fEPSP amplitudes at the 25th and 125th stimulus of the first stimulation train for individual WT (blue dots, 11 slices from 5 animals) and SynTKO (red dots, 12 slices from 6 animals) recordings. Median values ± interquartile ranges are shown in black. Ranks were not significantly different for either 25th (*p* = 0.695; Mann–Whitney *U* test) or 125th stimulus (*p* = 0.74, Mann–Whitney *U* test). ***d***, Normalized fEPSP amplitudes at the 25th and 125th stimulus of the fourth stimulation train for individual WT (blue dots, 11 slices from 5 animals) and SynTKO (red dots, 12 slices from 6 animals) recordings. Median values ± interquartile ranges are shown in black. Ranks were significantly different at the 125th stimulus (*p* = 0.032; Mann–Whitney *U* test), but not at the 25th stimulus (*p* = 0.88; Mann–Whitney *U* test). ***e***, The loss of fibers during high-frequency stimulation is not substantial and similar for SynTKO and WT mice. Exemplary traces from high-frequency stimulation trains for WT (blue) and SynTKO (red) animals. The 10th PFV and fEPSP from the first and fourth stimulation train are depicted, respectively. Dotted lines indicate the peaks of the PFV. Note that although the PFV is smaller for SynTKO (due to technical reasons in response to the high excitability), the size is relatively consistent throughout the trains. ***f***, Averaged PFV (mV) taken from 10–15 pulses from the first and fourth stimulation train, respectively, for recordings from WT (blue, 11 slices from 5 animals) and SynTKO (red, 12 slices from 6 animals) slices. Average values from the same recording are connected. Median values and interquartile ranges are depicted in black. Ranks between first and fourth stimulation train were not significantly different for neither WT (*p* = 0.36) nor SynTKO (*p* = 0.08) recordings, compared in a Wilcoxon test. ***g***, The relative loss of fibers was similar for WT and SynTKO recordings. Averaged ratios between fourth and first train PFV sizes are depicted for both WT (blue, 11 slices from 5 animals) and SynTKO (red, 12 slices from 6 animals) animals. Median values and interquartile ranges are depicted in black. Ranks were not significantly different (*p* = 0.24) in a Mann–Whitney *U* test.

**Figure 3.**
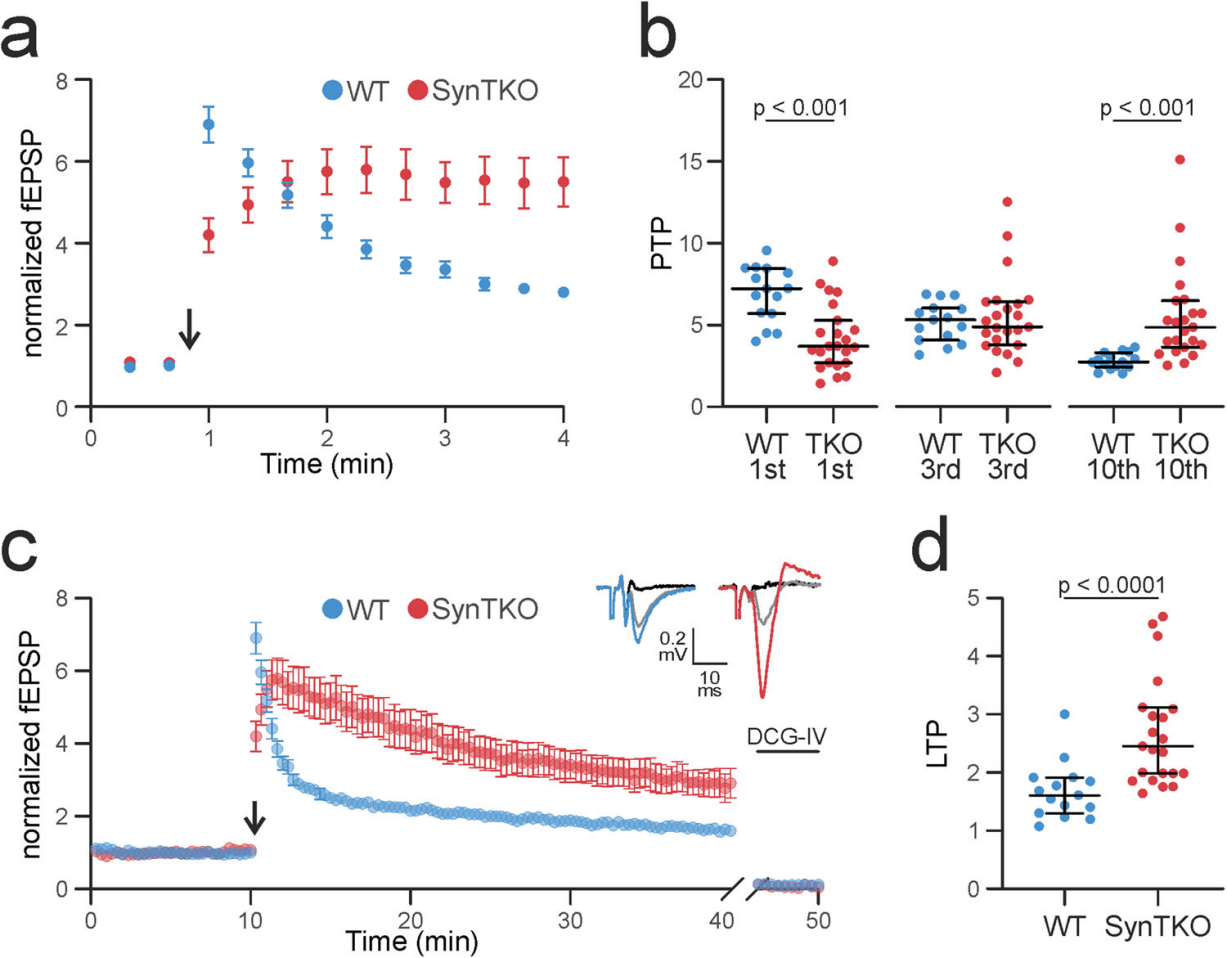
SynTKO mossy fibers display reduced post-tetanic potentiation but increased long-term potentiation. ***a***, Post-tetanic potentiation is decreased in SynTKO mossy fibers. Normalized averaged fEPSP amplitudes plotted against time (min) from WT (blue, 15 slices from 6 animals) and SynTKO (red, 23 slices from 9 animals) recordings. This plot is a partial zoom-in from the plot shown in ***c*** (9–13  min). Mean values ± SEM are shown. The arrow indicates the time point of high-frequency stimulation (4 times 125 pulses at 25  Hz). Stimulation before and after was at 0.05  Hz. Statistics for dataset as reported in ***c***. ***b***, Scatterplots for individual fEPSP amplitudes for WT (blue) and SynTKO (red) recordings for the 1st, 3rd, and 10th stimulus after high-frequency stimulation, respectively. Median values ± interquartile ranges are shown in black. The significance was tested with a Kruskal–Wallis test and a post hoc Dunn’s correction for multiple comparisons. The Kruskal–Wallis test revealed significant differences between ranks with *p* < 0.0001. Multiple comparisons revealed significant differences for the 1st (*p* = 0.0003) and 10th (*p* = 0.0002) time point. ***c***, LTP is increased in SynTKO animals after 30  min. Top, Example traces of fEPSP amplitudes 30  min after high-frequency stimulation for mossy fibers from WT (blue, left) and TKO (red, right) mice compared with baseline fEPSP amplitude (gray) and response to 1  µM DCG-IV (black). Bottom, Normalized averaged fEPSP amplitudes plotted over time (min) from WT (blue, 15 slices from 6 animals) and SynTKO (red, 23 slices from 9 animals) recordings. Mean values ± SEM are shown. The arrow indicates the high-frequency stimulation (4 times 125 pulses at 25  Hz). Stimulation frequency before (baseline) and after (LTP recording) was 0.05  Hz. At the end of the recording, 1  µM DCG-IV was washed in to ensure mossy fiber specificity. The last 10 fEPSP amplitudes during DCG-IV wash-in are shown at the end of the recording. A mixed-effect model revealed significant differences for the genotype (*p* = 0.005), time (*p* < 0.0001), and the interaction of both (*p* < 0.0001). A post hoc Sidak’s test for multiple comparisons revealed significant differences for the first sweep after high-frequency stimulation (*p* = 0.0125) and Sweeps 38 (*p* < 0.05) and Sweeps 40–54 (*p* < 0.05; ∼14–18  min), as well as for Sweeps 61, 67, and 94 (*p* < 0.05; ∼20, 22, and 32  min). ***d***, Dots indicate averaged fEPSP amplitudes from individual WT (blue) and SynTKO (red) recordings. Amplitudes were averaged over the last 10  min of the LTP recording; from 20 to 30  min after high-frequency stimulation. Median values ± interquartile ranges are shown in black. Ranks were significantly different with *p* < 0.0001 (Mann–Whitney *U* test).

For Figure 1*a,e*, data points were fitted with a simple linear regression. Slopes of those regressions were tested with a two-tailed ANCOVA and are shown with 95% confidence bands. For Figures 1*b*,*f*, 2*a*,*b*, and 3*a*,*c*, data were tested with a mixed-effects model and a post hoc Sidak's test for multiple comparisons. Factors time, genotype, and the interaction of both were tested. For Figures 1*c*,*d*,*g*,*h*, 2*c*,*d*,*g*, and 3*d*, ranks were compared with Mann–Whitney *U* tests. For data in Figure 3*b*, we used a Kruskal–Wallis test with a post hoc Dunn's correction for multiple comparisons. For Figure 2*f* we used a Wilcoxon test to compare ranks.

#### R project for statistical computing in RStudio

**Figure 4.**
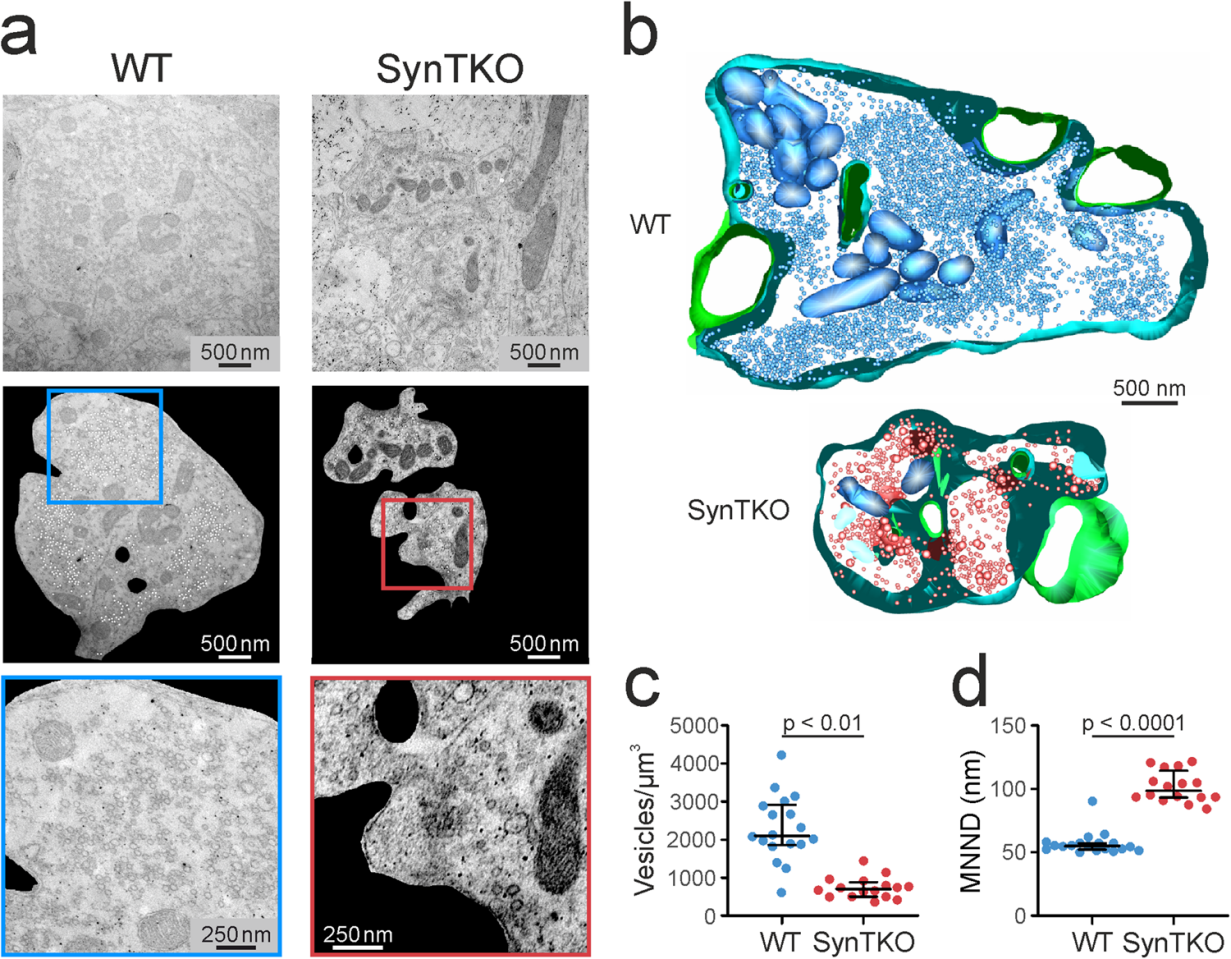
Synaptic vesicles are more dispersed in mossy fiber boutons from SynTKO mice. ***a***, In mossy fiber boutons, synaptic vesicles are more dispersed, and their density is reduced. Example images from TEM showing mossy fiber boutons from WT (left) and SynTKO (right) animals. Top, Raw TEM images of mossy fiber boutons in stratum lucidum. Middle, An automated tool (eImbrosci et al., 2022) was used to detect vesicles. Mossy fiber boutons were extracted from the raw image, and the center of detected vesicles is marked with a white dot. Blue and red boxes show the region for the zoom-ins in WT and SynTKO, respectively. Bottom, Zoom-ins, as marked in the middle pictures. High-magnification images of mossy fiber boutons from a WT and a SynTKO animal, respectively. Note the reduced abundance of synaptic vesicles in the SynTKO bouton. ***b***, Partial 3D reconstruction of hippocampal mossy fiber boutons from a WT (top) and a SynTKO animal (bottom) for visualization purposes only. Vesicles are shown in blue and red, respectively, the presynaptic mossy fiber membrane is shown in light blue, and postsynaptic spines are shown in green. ***c***, The number of synaptic vesicles per cubic micrometer is reduced in SynTKO animals. Dots represent the number of vesicles in individual mossy fiber boutons from three WT (blue, 18 boutons) and three SynTKO (red, 16 boutons) animals. Median values and interquartile ranges are shown in black. A generalized linear mixed model revealed significant differences between genotypes with *p* = 0.0015. ***d***, The MNND is increased between synaptic vesicles in SynTKO compared with those in WT boutons. The scatterplot shows average MNND (nm) for individual mossy fiber boutons from three WT (blue, 18 boutons) and three SynTKO (red, 16 boutons) animals. Genotypes were significantly different in a generalized linear mixed model with *p* = 0.0015. Median values are shown in black with interquartile ranges.

**Figure 5.**
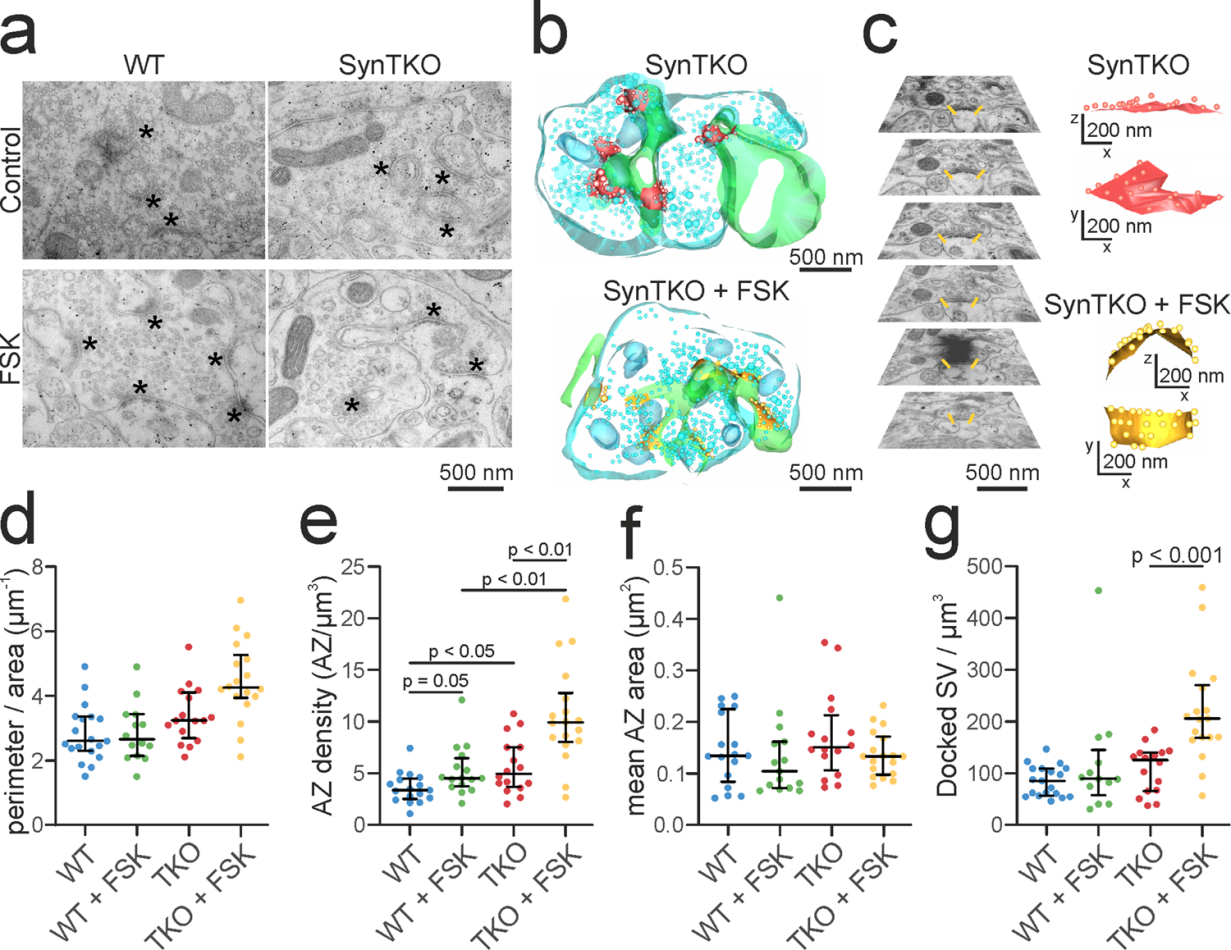
Increased active zone density in mossy fiber boutons of SynTKO mice. ***a***, Example images from TEM showing mossy fiber boutons from WT (left) and SynTKO (right) animals in control (top) and FSK (bottom) condition. Black asterisks indicate the active zones. ***b***, Example partial 3D reconstructions of mossy fiber boutons from untreated (top) and FSK-treated (bottom) SynTKO mice. Active zones with docked vesicles are shown in red (SynTKO) and yellow (SynTKO + FSK), respectively. Synaptic vesicles, mitochondria, and presynaptic membrane are shown in light blue; the postsynaptic membrane is shown in green. ***c***, Single active zones were reconstructed from serial sections of TEM images. Left, An example stack of serial sections for one active zone, indicated by yellow lines at the active zone boundaries. Right, 3D reconstructions of single active zones from untreated (red) and FSK-treated (yellow) SynTKO mice, in side and top view, respectively. The yellow active zone corresponds to the serial images to the left. ***d***, Complexity of boutons [measured as perimeter/area (µm^-1^)] plotted for individual mossy fiber boutons from untreated WT (blue dots, 17 boutons from 3 animals) and untreated SynTKO slices (red dots, 16 boutons from 3 animals) as well as for FSK-treated WT (green dots, 16 boutons from 3 animals) and FSK-treated SynTKO slices (yellow dots, 18 boutons from 3 animals). Median values are shown in black with interquartile ranges. A hypothesis test between generalized linear mixed models revealed no significant differences (*p* = 0.3972). ***e***, The number of active zones per cubic micrometer plotted for individual mossy fiber boutons from untreated WT (blue dots, 17 boutons from 3 animals) and untreated SynTKO slices (red dots, 16 boutons from 3 animals) as well as for FSK-treated WT (green dots, 16 boutons from 3 animals) and FSK-treated SynTKO slices (yellow dots, 18 boutons from 3 animals). Median values are shown in black with interquartile ranges. A hypothesis test between nested generalized linear mixed models revealed significant differences (*p* = 0.04). A post hoc test (marginal contrasts analysis with *p* value adjustment) revealed significant differences between WT and SynTKO (*p* = 0.0359), WT and WT + FSK (p = 0.05), SynTKO and SynTKO + FSK (*p* = 0.005), and WT + FSK and SynTKO + FSK (*p* = 0.005), but no significant difference between WT + FSK and SynTKO (*p* = 0.772). ***f***, The mean active zone area (µm^2^) per bouton for individual mossy fiber boutons from untreated WT (blue dots, 17 boutons from 3 animals) and untreated SynTKO slices (red dots, 16 boutons from 3 animals) as well as for FSK-treated WT (green dots, 16 boutons from 3 animals) and FSK-treated SynTKO slices (yellow dots, 18 boutons from 3 animals). Median values and interquartile ranges are shown in black. A hypothesis test between generalized linear mixed models revealed no significant differences (*p* = 0.8112). ***g***, The number of docked synaptic vesicles per cubic micrometer plotted for individual mossy fiber boutons from untreated WT (blue dots, 17 boutons from 3 animals) and untreated SynTKO slices (red dots, 16 boutons from 3 animals) as well as for FSK-treated WT (green dots, 16 boutons from 3 animals) and FSK-treated SynTKO slices (yellow dots, 18 boutons from 3 animals). Median values are shown in black with interquartile ranges. A hypothesis test between generalized linear mixed models revealed significant differences for FSK treatment (*p* = 0.0002). A post hoc test (marginal contrasts analysis with *p* value adjustment) revealed significant differences between SynTKO and SynTKO + FSK (*p* = 0.0009).

One of the central assumptions of many statistical tests is independence of data points, which is not given in a nested experimental design as ours (Galbraith et al., 2010; Krzywinski et al., 2014). Nested structures introduce correlations in the sample, which have to be accounted for, to avoid inflation of false-positive conclusions (Lazic, 2010; Aarts et al., 2014). To account for the multilevel nested structure of the electron microscopy data (Figs. 4*c*,*d*, 5*d*–*g*), we wrote generalized linear mixed models from the gamma family with a log link. We used the glmer function from the R package: lme4 (RRID:SCR_015654; Bates et al., 2015) to fit the generalized linear mixed models. Nested models were compared with likelihood ratio tests for testing different hypotheses. In case of the data for Figure 5*e*,*g*, we performed post hoc tests (marginal contrasts analysis) for multiple comparisons. For obtaining and contrasting the marginal means, we used the R package: emmeans (RRID:SCR_018734). *P* values were adjusted using a false discovery rate correction (Benjamini and Hochberg, 1995). The scripts for the statistical analysis and the corresponding data tables are available on GitHub: https://github.com/FeliBrue/Bruentgens_et_al_2024.

## Results

### Mossy fibers of SynTKO animals are more excitable

Despite the sparse connectivity (Amaral et al., 1990) and the low baseline activity of granule cells (Jung and McNaughton, 1993), a single mossy fiber bouton is able to trigger the discharge of its postsynaptic partner (Henze et al., 2002; Vyleta et al., 2016). Mossy fiber activity is not only important for pattern separation in the healthy brain (Rolls, 2018) but also for propagation of seizures in the epileptic brain (Nadler, 2003). Since SynTKO animals display high network excitability and develop epileptic seizures at the age of two months (Gitler et al., 2004; Fassio et al., 2011), we tested the excitability in mossy fibers by measuring the input–output relationship. We performed experiments in presymptomatic (4–6 weeks old) and symptomatic (17–19 weeks old) animals, after the onset of epileptic seizures. This design was aimed at differentiating changes in synaptic transmission that could lead to or result from epilepsy in SynTKO animals.

We conducted field recordings in acute slices from SynTKO and WT age-matched controls and recorded the input–output relationship as a measure of synaptic strength. We recorded from the *stratum lucidum* of area CA3 while stimulating close to the granule cell layer in the hilus. We found that SynTKO were significantly more excitable than WT animals: the input–output relation was increased in both 4–6 weeks old (Fig. 1*a*) and 17–19 weeks old animals (Fig. 1*e*). With the same amount of stimulated fibers (size of the PFV), the fEPSP amplitudes were larger. The slopes of the simple linear regression fits of fiber volley versus fEPSP amplitudes were significantly different with *p* < 0.0001 between control and SynTKO data for both age groups. For presymptomatic recordings, the slopes for the simple linear regression with 95% confidence intervals were 0.083 [0.005–0.171] for WT and 2.743 [2.159–3.326] for SynTKO and for symptomatic recordings 0.795 [0.59–0.99] for WT and 2.292 [1.806–2.777] for SynTKO.

Changes at the network level, morphological changes, or changes in release probability could underlie this increased excitability. At the network level, this could be explained by the fact that the lack of synapsins differentially affects excitatory and inhibitory neurons (Farisello et al., 2013). Morphology was described to be similar between WT and SynTKO animals (Gitler et al., 2004); therefore we assume that the number of excitable fibers is comparable. To check for a possible change in release probability, we measured the PPR with an interstimulus interval of 50 ms. Under our experimental conditions, the PPR was not significantly different between presymptomatic SynTKO and WT animals (Fig. 1*d*). In WT recordings, the median PPR was 2.827 [2.423; 3.795], while in recordings from SynTKO, it was 3.745 [2.422; 5.570] (*p* = 0.133; Mann–Whitney U test). However, we saw a trend for an increased PPR that became clearer with a shortening of the interstimulus interval to 40 ms (see first two fEPSPs in Fig. 2*a*,*b*). Here, in WT recordings, the median PPR was 2.91 [2.654; 4.026], while it was 4.436 [3.485; 6.094] for SynTKO. The *p* value was 0.07 (Mann–Whitney U test). Finally, in symptomatic SynTKO animals, the PPR was significantly increased compared with WT animals with *p* = 0.03 (Mann–Whitney U test) with a median of 2.906 [2.549; 3.499] for WT and 3.435 [2.964; 4.944] for SynTKO animals (Fig. 1*h*). A change in PPR is suggestive of a change in release probability (Dobrunz and Stevens, 1997); however, this might be taken with caution as many other factors affect this measure (Hanse and Gustafsson, 2001; H. Y. Sun et al., 2005; Neher and Brose, 2018; Glasgow et al., 2019).

### Reduced frequency facilitation in mossy fibers of SynTKO animals

Mossy fiber boutons are very powerful synapses when it comes to presynaptic plasticity. They are able to facilitate dramatically, even at moderate frequencies (Salin et al., 1996). This phenotype, together with large pools of synaptic vesicles (Hallermann et al., 2003; Rollenhagen et al., 2007), makes them an excellent system for studying the influence of synapsins on presynaptic plasticity. In previous work, it has been reported that frequency facilitation is impaired at mossy fibers from SynDKO animals after stimulation with a moderate frequency of 2 Hz (Owe et al., 2009). The authors suggested that the remaining synapsin isoform—SynIII—causes impaired facilitation since it was localized in the RRP of mossy fiber boutons. Additionally, neurons from SynIII KO animals show less synaptic depression than WT neurons in primary hippocampal cultures (Feng et al., 2002). Here, we intended to test if complete deletion of synapsins, including SynIII, would rescue frequency facilitation at the hippocampal mossy fiber bouton.

When stimulated with a train of 20 pulses at a frequency of 1 Hz, we saw less facilitation in mossy fibers from presymptomatic SynTKO compared with WT animals. This finding is comparable to the aforementioned experiments in SynDKO animals (Owe et al., 2009) and cell culture experiments of SynTKO animals (Gitler et al., 2004). The rise in the field excitatory postsynaptic potential (fEPSP) amplitudes was similar in WT and SynTKO during the first 10 stimuli, but in SynTKO animals we observed an earlier saturation of amplitudes (Fig. 1*b*). When comparing the plots with a mixed-effects model, we found significant differences for the factor time (*p* < 0.0001), as well as for the interaction between time and genotype (*p* < 0.001). The post hoc Sidak's test for multiple comparisons revealed no significant differences for single time points (*p* > 0.05). When comparing the amplitudes in response to the last 1 Hz stimulus, we found that the median facilitation was 6.52 [5.553; 9.047] for WT animals, while the increase was only 5.110 [3.76; 7.08] compared with the baseline for SynTKO animals [median value (25% quartile; 75% quartile)]. Ranks were different with *p* = 0.0089 [Mann–Whitney* U* test (Fig. 1*c*)].

All synapsin KO animals lacking SynI and/or SynII develop seizures beginning at the age of two months (Fassio et al., 2011) that could potentially lead to secondary differences in plasticity. We therefore investigated short-term plasticity also in 17–19-week-old symptomatic mice—matching the age range from Owe et al. (2009). In symptomatic mice, we observed a more pronounced effect on frequency facilitation (Fig. 1*f*,*g*): the facilitation in WT animals reached 7.019 [5.574; 8.440], while the increase in SynTKO recordings was only 4.414 [4.036; 5.330] compared with the baseline [median (25% quartile; 75% quartile)]. Ranks were significantly different with *p* = 0.0009 (Mann–Whitney *U* test). In summary, we see a decrease in frequency facilitation but an increase in excitability in SynTKO animals. These results indicate that (1) the presence rather than the absence of specific synapsin isoforms is needed to rescue facilitation and (2) excitability and short-term plasticity mechanisms are already altered before the onset of epileptic seizures.

### High-frequency stimulation leads to early vesicle exhaustion in SynTKO animals

Since stimulation with a moderate frequency led to a decrease in facilitation in presymptomatic SynTKO animals (Fig. 1*b*,*c*), we wanted to investigate the response to a longer stimulation with a higher frequency. We applied four trains of 125 pulses at 25 Hz. While the course of the amplitudes was very similar in the first high-frequency train for both genotypes (Fig. 2*a*,*c*), changes manifested over time. Differences between WT and SynTKO animals became distinguishable in the fourth stimulation train (Fig. 2*b*,*d*) with smaller amplitudes towards the end of the train in SynTKO animals.

When tested with a mixed-effects model, we detected significant differences for the factor time (stimuli) for both the first and the fourth stimulation train (*p* < 0.0001). A post hoc Sidak's test for multiple comparisons revealed no significant differences for single time points for either of the stimulation trains. We also compared the amplitudes for the 25th and 125th stimulus of the stimulation trains between genotypes. Ranks were comparable between genotypes in the first stimulation train at the 25th and 125th stimulus as well as at the 25th stimulus of the fourth stimulation train (Table 7). However, when comparing the amplitudes of the 125th stimulus of the fourth stimulation train, we found a significant difference (*p* = 0.032, Mann–Whitney *U* test) between WT and SynTKO (Fig. 2*d*). Here, the median normalized fEPSP amplitude was 0.687 [0.501; 1.987] for WT and 0.3714 [0.049; 0.72] for SynTKO animals. This stronger exhaustion during intense stimulation was already described before in other synapsin KO animals (Rosahl et al., 1995; Farisello et al., 2013) and probably reflects the missing reserve pool, which would normally replenish the RRP under such high activity (Vasileva et al., 2012).

**Table 7.** Normalized averaged fEPSP amplitudes during high-frequency stimulation at 25 Hz

Train	Measure	WT animals	SynTKO animals
First train 25th stimulus	Median	3.704	3.653
	Interquartile range	[1.873; 7.935]	[2.426; 7.092]
	*p* value (Mann–Whitney *U* test)	*p* = 0.695	
First train 125th stimulus	Median	1.243	0.7514
	Interquartile ranges	[0.473; 2.332]	[0.4997; 1.650]
	*p* value (Mann–Whitney *U* test)	*p* = 0.74	
Fourth train 25th stimulus	Median	3.176	1.61
	Interquartile ranges	[1.039; 3.77]	[0.75; 4.314]
	*p* value (Mann–Whitney *U* test)	*p* = 0.88	
Fourth train 125th stimulus	Median	0.687	0.3714
	Interquartile ranges	[0.501; 1.987]	[0.049; 0.72]
	*p* value (Mann–Whitney *U* test)	*p* = 0.032	

Amplitudes were measured in both genotypes in the first and fourth train of 125 pulses at 25 Hz (Fig. 2) at the 25th and 125th stimulus, and their ranks were compared in Mann–Whitney *U* tests.

High-frequency stimulation can lead to a loss of fibers during the course of stimulation. To check if the smaller fEPSP amplitudes in the last stimulation train of SynTKO recordings are due to an increased fiber loss, we measured a subset of the PFVs during the first and fourth stimulation train for both genotypes, respectively. While PFVs of SynTKO animals were in general smaller than the ones from WT animals with comparable fEPSP amplitudes (Fig. 1*a*), there was no relative difference in PFV sizes of the two genotypes between first and last stimulation train (Fig. 2*e*–*g*). Thus, we conclude that the relative loss of fibers is similar for both genotypes and does not explain the more drastic decrease in the fEPSP size for SynTKO animals.

Here, our data indicate that deletion of all synapsins disturbs vesicle organization in synaptic terminals in a way that leads to impaired replenishment. This is especially relevant for synapses like mossy fiber boutons, which have large vesicle pools (Hallermann et al., 2003; Rollenhagen et al., 2007).

### PTP is changed in SynTKO animals

After intense stimulation of mossy fibers, PTP (Griffith, 1990), another form of short-term plasticity, occurs, which was proposed to underlie short-term memory (Vandael et al., 2020). Measuring PTP after four trains of high-frequency stimulation revealed differences between WT and SynTKO animals: while in WT recordings the median potentiation was 7.229 [5.701; 8.464]-fold compared with the baseline and decreased over time, in SynTKO recordings, we initially measured an amplitude which was only 3.702 [2.683; 5.280] times larger than the baseline (significantly different in a Kruskal–Wallis test with post hoc Dunn's test for multiple comparisons; *p* = 0.0003) but increased over time. After 1 min, the amplitudes of WT and SynTKO recordings were comparable (Fig. 3*a*,*b*; WT, 5.323 [4.070; 6.03]; SynTKO, 4.887 [3.769; 6.419]; *p* > 0.99), followed by a further increase in the SynTKO amplitudes over WT amplitudes. One minute after stimulation, the amplitudes of the SynTKO animals remained on a plateau, while the amplitudes in the WT animals decreased further (Fig. 3*a*,*b*), leading to median amplitudes of 2.749 [2.432; 3.321] for WT and 4.865 [3.635; 6.497] for SynTKO animals ∼3 min after high-frequency stimulation (significantly different with *p* = 0.0002). These findings point to different underlying mechanisms: one leading to the impairment of PTP right after high-frequency stimulation and another one leading to increased amplitudes after some recovery time and upon low-frequency stimulation of 0.05 Hz. To understand this observation further, we continued recording for half an hour, which corresponds to early LTP.

### LTP is enhanced in SynTKO animals

Mossy fiber boutons express a presynaptic form of LTP, which is PKA-dependent (Weisskopf et al., 1994). In recordings from SynDKO animals, mossy fiber LTP was unchanged compared with WT animals (Spillane et al., 1995). It is tempting to speculate, though, that SynIII might be the phosphorylation target of PKA in the context of LTP, since a PKA phosphorylation site is present in domain A (Piccini et al., 2015), which is conserved among all synapsins, and SynIII expression is maintained in adult mossy fiber boutons (Pieribone et al., 2002). A complete loss of synapsins could therefore lead to a block of LTP. However, when recording LTP (Fig. 3*c*), we measured a median potentiation of 1.6 [1.3; 1.92] in WT animals 20–30 min after the high-frequency stimulation, while SynTKO animals showed a larger median potentiation of 2.45 [1.98; 3.12] compared with the baseline (Fig. 3*d*). Ranks differed significantly with *p* < 0.0001 (Mann–Whitney *U* test). The time course of LTP was tested in a mixed-effects model. The factors genotype, time, and the interaction of both differed significantly (*p* < 0.0001; *p* = 0.005; *p* < 0.0001, respectively). A post hoc Sidak's test for multiple comparisons revealed significant differences for single time points as well (Fig. 3*c* legend). We included all measurements that fulfilled the specificity criterion, which was tested by the application of the metabotropic glutamate receptor group II agonist DCG-IV (Kamiya et al., 1996; last 10 sweeps are shown in Fig. 3*c*).

In summary, the absence of all synapsin isoforms in mossy fiber synapses leads to a reduced early PTP, an altered time course of PTP/LTP and an increased long-lasting potentiation. Such changes in LTP have not been described before in other synapsin KO models, suggesting that this effect is specifically relevant for the mossy fiber bouton, where LTP occurs presynaptically (Zalutsky and Nicoll, 1990) and only present upon the complete loss of synapsins. Since it has been shown that ultrastructural changes underlie potentiation at hippocampal mossy fibers (Orlando et al., 2021), we next sought to investigate the ultrastructure of mossy fiber boutons in SynTKO animals.

### Synaptic vesicles are more dispersed in SynTKO animals

So far, vesicle distributions at the hippocampal mossy fiber bouton have only been described for either SynDKO animals or SynIII KO animals (Feng et al., 2002; Owe et al., 2009). Here, we wanted to test whether the KO of all three synapsins would lead to additional changes in vesicle organization at the hippocampal mossy fiber bouton. Using TEM, we identified individual mossy fiber boutons from three presymptomatic SynTKO and three age-matched WT mice. We imaged serial sections from 16 SynTKO and 18 WT mossy fiber boutons. For each 2D projection, we measured the vesicle number and the MNND using an automated tool (Imbrosci et al., 2022; Fig. 4). For both datasets, we used a generalized linear mixed model to estimate either the vesicle density or MNND. When comparing synaptic vesicles of WT and SynTKO boutons, the median density was strongly reduced in boutons from SynTKO animals (702.5 [499; 882.9] vesicles/µm^3^ compared with 2,102 [1,861; 2,916] vesicles/µm^3^ in WT; Fig. 4*b*,*c*). In a hypothesis test between nested models, the genotypes were significantly different with *p* = 0.0015. Consequently, we also saw an increase in the MNND of vesicles (Fig. 4*b,d*): the median MNND was 55.05 [52.24; 57.28] nm for WT and 98.58 [93.38; 114.4] nm for SynTKO boutons. Groups were significantly different with *p* = 0.0015. The reduced density of distal vesicles implies a reduced reserve pool. Since this observation resembles the results seen in mossy fiber boutons of SynDKO animals (Owe et al., 2009), our data indicate that the additional KO of SynIII does not add on effects on the organization of the distal pool. This conclusion is also in line with unchanged synaptic vesicle densities in mossy fiber boutons of SynIII KO mice (Feng et al., 2002).

Together, our data confirm a reduced distal vesicle pool, as it had been described before in cultured SynTKO and SynDKO mossy fibers (Spillane et al., 1995; Siksou et al., 2007).

### Active zone density is highest in chemically potentiated mossy fiber boutons from SynTKO animals

Since we saw an increase in LTP in SynTKO animals (Fig. 3*c*,*d*), we wanted to understand if structural changes would occur in potentiated mossy fiber boutons from SynTKO animals. We performed TEM in hippocampal slices from young WT and presymptomatic SynTKO animals in either potentiated or control conditions. Potentiation was chemically induced via incubation with the adenylyl cyclase-activator FSK before fixation of the samples. FSK induces an increase in intracellular cAMP and has similar effects on mossy fibers as high-frequency electrical stimulation (Weisskopf et al., 1994; Spillane et al., 1995). Structural measures following potentiation included bouton complexity, active zone density, active zone area, and docked synaptic vesicle density. We estimated bouton complexity as the ratio between perimeter and area of each presynaptic profile in 2D images from three animals per group. The median complexity was similar between control and chemically potentiated boutons of both WT and SynTKO animals [median value (interquartile range) for WT: 2.61 [2.29; 3.35] µm^−1^, for WT + FSK: 2.65 [2.13; 3.43] µm^−1^, for SynTKO: 3.24 [2.69; 4.09] µm^−1^ and for SynTKO + FSK: 4.25 [3.93; 5.25] µm^−1^; Fig. 5*d*]. A hypothesis test between nested generalized linear mixed models revealed no significant differences (*p* = 0.3972).

The active zone density was analyzed in partial 3D reconstructions of mossy fiber boutons as the measure of the total number of reconstructed active zones (see Materials and Methods for details) normalized by the volume of the reconstructed bouton (µm^3^). We fitted a generalized linear mixed model to estimate active zone density given the genotype and FSK treatment. We found a significant difference for FSK treatment (*p* < 0.001) when we compared the model with the respective null model. Specific pairs were compared by testing estimated marginal means with adjustment for the false discovery rate (Benjamini and Hochberg, 1995).

We observed a significant increase (*p* = 0.0134) in the active zone density in WT animals when treated with FSK, as described before (Orlando et al., 2021). Untreated boutons from SynTKO animals had a similar mean density of active zones as FSK-treated boutons from WT animals (5.63 [4.43; 7.14] active zones/µm^3^ for untreated SynTKO boutons; 5.22 [4.13; 6.60] active zones/µm^3^ for FSK-treated WT boutons, *p* = 0.658). This indicates that, from a structural point of view, SynTKO animals could be in a similar state as potentiated WT boutons. Treatment with FSK led to a further increase in the active zone density in mossy fiber boutons from SynTKO animals (10.20 [7.50; 13.88] active zones/µm^3^; Fig. 5*a*,*b*) and led to significant differences when compared with untreated SynTKO boutons (*p* = 0.0018) as well as treated WT boutons (*p* = 0.0018; Fig. 5*e*).

Our previous work revealed that the FSK-induced structural changes were not accompanied by a change in the active zone area of mossy fiber boutons (Orlando et al., 2021). To test if this holds true in SynTKO animals, we analyzed the area of individual active zones from partial 3D reconstructions of treated and untreated WT and SynTKO animals. Mean active zone areas per bouton were comparable for both genotypes and treatments (Fig. 5*f*), with median areas of 0.13 [0.08; 0.22] µm^2^ for WT, 0.1 [0.07; 0.16] µm^2^ for WT + FSK, 0.15 [0.1; 0.2] µm^2^ for SynTKO, and 0.13 [0.09; 0.17] µm^2^ for SynTKO + FSK. We fitted a generalized linear mixed model to estimate the active zone area given the genotype and treatment. When comparing the model to a nested null model, we found no evidence for a significant difference between models (*p* = 0.8112), indicating no differences in the active zone areas.

### RRP correlate increases in chemically potentiated SynTKO mossy fiber boutons

Finally, we wanted to assess if the number of docked vesicles would change depending on the genotype or treatment. While chemical fixation is not ideal for the analysis of this parameter, our analysis allows us to get an idea of potential changes in the number of docked vesicles. We analyzed the number of docked vesicles per 3D reconstruction and normalized the total number to the respective bouton volume. The docked synaptic vesicle density was slightly increased from control to FSK-treated WT boutons with a median density of 85.36 [56.39; 108.8] synaptic vesicles/µm^3^ for WT and 89.75 [57.54; 145.1] synaptic vesicles/µm^3^ for WT + FSK. In contrast, the docked synaptic vesicle density increased in FSK-treated SynTKO boutons when compared with that in untreated SynTKO: the median density was 125.5 [65.69; 139.6] synaptic vesicles/µm^3^ for SynTKO and 205.7 [168.8; 270.5] synaptic vesicles/µm^3^ for SynTKO + FSK. We fitted a generalized linear mixed model to estimate the normalized number of docked vesicles given the genotype and treatment. When comparing the model to nested null models, we found a small—not statistically significant—increase in docked vesicles in FSK-treated WT boutons (*p* = 0.08) and a significant effect of FSK treatment on the number of docked vesicles in the SynTKO animals (*p* = 0.0009; Fig. 5*g*).

Taken together, our data show a structural strengthening in boutons from SynTKO animals: both the RRP correlate and the active zone density (Fig. 5) are increased upon FSK treatment, which might explain the increase in LTP (Fig. 3*c*,*d*).

## Discussion

Here, we investigated the role of synapsin in various forms of presynaptic plasticity at a glutamatergic synapse that retains SynIII expression in adulthood. The genetic deletion of SynI, SynII, SynIII, SynI/II, and SynI/II/III has been investigated extensively in culture and in various synapses from different brain regions over the last years (see Table 8 for an overview). Despite the extensive work, hippocampal mossy fiber plasticity of mice lacking all synapsin isoforms was not yet characterized. To fill this knowledge gap, we performed local field recordings and 3D electron microscopy at hippocampal mossy fibers from SynTKO and age-matched WT male mice. The removal of all synapsin isoforms from hippocampal mossy fiber boutons leads to a phenotype that recapitulates previously published data (Table 8): the dispersion of synaptic vesicles out of the bouton and impaired short-term plasticity. The impaired PTP indicates a potential role of synapsins in short-term memory. We additionally observed increased LTP in SynTKO mossy fibers, accompanied by an increase in active zone density. Together, our results show that synapsins play a role in the modulation of mossy fiber-specific presynaptic plasticity.

**Table 8.** Overview of functional and structural changes in glutamatergic synapses of synapsin KOs

Genotype	Syn I KO	Syn II KO	Syn III KO	SynDKO	SynTKO	References
Excitability		↑			↑ **(↑)**	Boido et al., 2010; Farisello et al., 2013; Matos et al., 2019 **(this work)**
Frequency facilitation	↓	=		**↓**	**(↓)**	[Bibr B64]**Owe et al., 2009**[Bibr B64]; Nikolaev and Heggelund, 2015 **(this work)**
Depression	=↑	=↑	↑	↑	↑ **(↑)**	Rosahl et al., 1995; Feng et al., 2002; Gitler et al., 2004; J. Sun et al., 2006; Vasileva et al., 2012; Farisello et al., 2013 **(this work)**
PTP	=↓	=↓		**↓**	↓ **(↓)**	Rosahl et al., 1993, 1995; [Bibr B82]**Spillane et al., 1995**[Bibr B82]; Kielland et al., 2006; Valente et al., 2012; Farisello et al., 2013; Nikolaev and Heggelund, 2015; Cheng et al., 2018 **(this work)**
LTP	**=**			**=**	**(↑)**	Rosahl et al., 1993, 1995; [Bibr B82]**Spillane et al., 1995**[Bibr B82]; [Bibr B86]**Takei et al., 1995**[Bibr B86] **(this work)**
Density of synaptic vesicles	**↓**		**=**	**↓**	↓**(↓)**	Li et al., 1995; Pieribone et al., 1995; Rosahl et al., 1995; [Bibr B86]**Takei et al., 1995**[Bibr B86]; [Bibr B22]**Feng et al., 2002**[Bibr B22]; Gitler et al., 2004; Siksou et al., 2007; [Bibr B64]**Owe et al., 2009**[Bibr B64]; Vasileva et al., 2012 **(this work)**

Arrows indicate an increase or decrease of the properties listed in the first column, while equal signs indicate no changes. Bold symbols indicate that previous work also considered the hippocampal mossy fiber bouton. References are marked accordingly. Arrows in brackets refer to the findings of this study.

In SynTKO mice, we found increased excitability, measured by a change in the input–output relation of local fEPSPs (Fig. 1). Although many factors can influence this measure, we think that a likely explanation is based on the finding that synapsins play different roles in excitatory versus inhibitory neurons (Song and Augustine, 2015). Deletion or mutation of SynI, SynIII, or all synapsins leads to impaired basal transmission of inhibitory, but not excitatory cultured neurons (Terada et al., 1999; Feng et al., 2002; Gitler et al., 2004; Baldelli et al., 2007). Loss of SynII impairs tonic inhibition in hippocampal slices (Medrihan et al., 2013, 2015) and increases excitability in hippocampal cultured neurons (Matos et al., 2019). Mossy fibers activate at least four times more inhibitory neurons than pyramidal cells in CA3 (Acsády et al., 1998), regulating CA3 excitability via feedforward inhibition (Acsády and Káli, 2007; Torborg et al., 2010). Reduced feedforward inhibition might thus explain the increased excitability. Indeed, the input–output relation is increased in Schaffer collaterals from SynTKO animals, while it is reduced in inhibitory fibers from CA1 (Farisello et al., 2013). Here we recorded extracellular local field potentials and could not address the contributing effects of the lack of synapsin in GABAergic synapses to the network.

During trains of activity, mossy fiber boutons facilitate reliably (Salin et al., 1996; Toth et al., 2000), a feature which is thought to be important for information transfer (Henze et al., 2002; Mori et al., 2004). In mossy fibers from SynDKO animals, frequency facilitation is reduced (Owe et al., 2009). Owe and coworkers suggested that the remaining SynIII may act as a brake on facilitation, because (1) SynIII is associated specifically with the RRP in mossy fiber boutons (Owe et al., 2009) and (2) synaptic depression is reduced in SynIII KO cultures (Feng et al., 2002). However, in animals lacking all synapsins, including SynIII, we still observed reduced frequency facilitation (Fig. 1). Frequency facilitation is most likely calcium-dependent and involves increased neurotransmitter release (Chamberland et al., 2017; Jackman and Regehr, 2017). Hence, potential reasons for reduced facilitation are diverse and include enhanced basal release probability, depletion of the RRP, and saturation of postsynaptic receptors (Neher and Sakaba, 2008).

High-frequency stimulation usually results in a biphasic depression, attributed to the depletion of the RRP (Zucker and Regehr, 2002) and slow replenishment from the reserve pool (Wesseling and Lo, 2002). We observed frequency-dependent depression for both genotypes when stimulating at 25 Hz (Fig. 2) but stronger depression in SynTKO animals, recapitulating previous results in SynTKO cultured neurons (Gitler et al., 2004). At the calyx of Held, a reduced reserve pool and slower replenishment accounted for faster depression in SynTKO animals (Vasileva et al., 2012). Indeed, in mossy fiber boutons of SynTKO animals, vesicles were reduced in density and more dispersed (Fig. 4), likely explaining faster depression. Impaired distal pools were described before for mossy fiber boutons (Takei et al., 1995; Owe et al., 2009) and in neuronal cultures from Syn KOs (Li et al., 1995; Gitler et al., 2004; Siksou et al., 2007), with the exception of SynIII KO mice (Feng et al., 2002; Table 8). Hence, at mossy fibers, the KO of all synapsins recapitulates previously described phenotypes of synaptic vesicle dispersion (Table 8). In general, vesicle declustering and reduced vesicle density likely have diverse effects on the release cycle (Bykhovskaia, 2011), possibly also supporting increased excitability and reduced frequency facilitation.

PTP has recently been suggested to underlie short-term memory. During mossy fiber PTP a “pool engram” is formed, i.e., the number of docked vesicles at active zones increases (Vandael et al., 2020). This engram formation depends on the refilling rate of vesicles and could thus be mediated by synapsins. In line with this hypothesis, we here show that the complete loss of synapsins impairs mossy fiber PTP significantly (Fig. 3). Reduced PTP was previously observed in synapsin KO models, with diversity regarding synapsin isoform and synapse type. PTP is slightly reduced (1) in mossy fibers of SynDKO mice (Spillane et al., 1995); (2) at Schaffer collaterals of SynI KO, SynII KO, SynDKO, and SynTKO mice (Rosahl et al., 1993, 1995; Farisello et al., 2013); (3) in cultured hippocampal neurons of SynI KO and SynTKO animals (Valente et al., 2012; Cheng et al., 2018); and (4) at corticothalamic synapses of SynI KO and SynDKO, but not SynII, KO animals (Kielland et al., 2006; Nikolaev and Heggelund, 2015). Here, we show a more drastic reduction in the initial PTP as well as an altered time course of PTP (Fig. 3) in comparison with SynDKO animals (Spillane et al., 1995), which leads us to hypothesize that also SynIII plays a role in mossy fiber presynaptic potentiation. Indeed, in cell culture, PTP measured via miniature excitatory postsynaptic currents could only be rescued by the SynIIIa isoform (Cheng et al., 2018). We could not test this hypothesis in this study due to the lack of direct comparison with SynIII knockouts.

We analyzed the density of docked vesicles as an ultrastructural correlate of the RRP and noticed no significant difference between SynTKO and WT mossy fiber boutons (Fig. 5*g*). Upon FSK treatment, we observed an increase in docked vesicles at SynTKO mossy fibers (Fig. 5*g*). We found a small (nonsignificant *p* = 0.08) increase in the RRP in WT boutons treated with FSK [in line with high pressure freezing experiments by Orlando et al. (2021) and Kim et al. (2023)]. This modulation of the vesicles close to active zones in SynTKO might indicate that synapsin-independent mechanisms are available at mossy fiber boutons that mobilize vesicles to the RRP upon increase in cAMP levels. Nevertheless, this dataset should not be overinterpreted: in fact, the use of glutaraldehyde for tissue preparation is thought to cause changes in docked vesicle measures due to its ability to cross-link proteins.

While the initial drop in PTP could be explained by impaired vesicle replenishment (Vasileva et al., 2012), we also observed a second, increased PTP phase (Fig. 3*a*,*b*). Alongside the RRP, also release probability and quantal size are increased during mossy fiber PTP (Vandael et al., 2020). Both could be elevated by default in SynTKO animals and increase PTP in the second phase. Interestingly, we detected an increase in active zone density and an increase in the RRP correlate upon FSK incubation in SynTKO boutons (Fig. 5*g*), which most likely reflects a change in the number of release sites. Hence, after overcoming the initial drop in PTP, other mechanisms could be untamed in SynTKO mossy fiber boutons, leading to enhanced PTP in a later phase.

The increased active zone density in SynTKO animals (Fig. 5*e*) could indicate a preset potentiated state (Orlando et al., 2021) due to homeostatic adaptation, similar to mechanisms in the calyx of Held of SynTKO animals (Vasileva et al., 2012). The active zone density was further increased when chemically potentiating SynTKO mossy fibers with FSK, leading to significantly higher densities than in FSK-treated WT boutons and untreated SynTKO boutons (Fig. 5*e*). An increase in the RRP correlate and active zone density after potentiation might also explain, to some extent, the increased LTP we observe in SynTKO animals (Figs. 3, 5*e,g*). Mossy fiber LTP has been analyzed previously in SynI KO (Takei et al., 1995) and SynDKO mice (Spillane et al., 1995) but was found to be unchanged. Thus, we speculate that the increase in LTP can only be detected upon the complete loss of synapsins.

Altogether our data suggest that, after overcoming the initial drop in PTP, other mechanisms could lead to enhanced PTP and LTP in SynTKO mossy fiber boutons.

It is unknown which mechanisms are shared between mossy fiber PTP and LTP. Synapsins might have specific functions in both processes, preventing excess release and balancing potentiation. Recent literature suggests that (1) diversity in STP depends on priming and fusion steps (Lin et al., 2022) and (2) increased fusion competence might underlie mossy fiber LTP, possibly mediated by Munc13-1 (Lipstein et al., 2021; Papantoniou et al., 2023; Fukaya et al., 2023a). Do synapsins—or specific synapsin isoforms—play a role in the regulation of vesicle docking, priming, and/or the insertion of new active zones at mossy fibers? Future work is needed to address these questions more specifically.

Here, we investigated plasticity at a glutamatergic synapse expressing SynIII in adulthood. We used SynTKO instead of SynIII KO animals to exclude compensatory effects via remaining synapsin isoforms. By combining physiological recordings—well-suited to record mossy fiber transmission (Breustedt et al., 2010)—and 3D ultrastructural analysis, our experiments shed light on synapsin-dependent plasticity from different angles. Our ultrastructural analysis is limited by the fact that chemical fixation is not the best method to investigate docked vesicles due to possible structural reorganization in the nanometer range, caused by glutaraldehyde-induced protein cross-linking. Another limitation is the limited volume of our partial 3D reconstruction. Future studies should utilize (1) high pressure freezing to draw more detailed conclusions regarding synapsins’ role in the pool engram regulation and (2) volume electron microscopy data to assess morphological changes in 3D reconstructions of whole boutons.

To exclude possible indirect estrogen effects on mossy fiber plasticity (Harte-Hargrove et al., 2013), we used male mice only, limiting the generalizability. Future studies should include female animals. Finally, although the chemical induction of mossy fiber potentiation using FSK is widely used, it is still unclear if it shares the same mechanisms as electrically induced potentiation (Shahoha et al., 2022; Fukaya et al., 2023b). The scope of this study was limited to the characterization of SynTKO and does not directly compare the structure and function of mossy fiber boutons in SynIII and SynI and II DKO. Further work is needed to dissect the precise role of the various synapsin isoforms both in hippocampal mossy fiber boutons and in other synapses (Pieribone et al., 2002).

In summary, our work revealed that the complete loss of synapsins leads to disruption of presynaptic plasticity at hippocampal mossy fibers. Facilitation and PTP are reduced, but LTP is increased, in concert with an elevated active zone density as well as an increased RRP correlate after FSK treatment. Our work contributes to a better understanding of mossy fiber presynaptic plasticity and, consequently, to a better understanding of synapsins’ roles in learning and memory.

## Data Availability

Data is fully available on request. Data tables and scripts for statistical analysis are available on GitHub: https://github.com/FeliBrue/Bruentgens_et_al_2024/. 

## Synthesis

Reviewing Editor: Viji Santhakumar, University of California Riverside Decisions are customarily a result of the Reviewing Editor and the peer reviewers coming together and discussing their recommendations until a consensus is reached. When revisions are invited, a fact-based synthesis statement explaining their decision and outlining what is needed to prepare a revision will be listed below. The following reviewer(s) agreed to reveal their identity: NONE. Note: If this manuscript was transferred from JNeurosci and a decision was made to accept the manuscript without peer review, a brief statement to this effect will instead be what is listed below.

The authors have address most major concerns abot data analysis in the revision and the mabuscript is much improved.

One issue that remains is a comprehensive assessment of the differences between the single KO in prior studies and the double knockout presented here. Specifically, the Discussion needs to clarify how the findings of this study differ from the results of earlier studies in single KOs. For example, changes in excitability in glutamatergic synapses (Fig. 1 A,B) were also found in Syn II KO. The reduced facilitation (Fig. 1 C) was observed in Syn I KO. The increased depression (Fig. 2 B) and SV depletion (Fig. 4 C) were observed in Syn II KO. The authors should explain which of the observed phenotypes were observed at single KO/DKO, which of them are additive for different Syn genes, and which are not additive. A table summarizing differences between genotypes would be helpful.

## Author Response

eNeuro eN-TNWR-0330-23X "The lack of synapsin alters presynaptic plasticity at hippocampal mossy fibers in male mice" Dear Prof. Grubb, Thank you for dedicating your time to reviewing our manuscript and for considering our work of potential interest.

We are grateful for the reviewers' comments and have followed their suggestions.

In summary, we performed new electrophysiological recordings to improve figures 1, 1-2, 2, 2-1 and 3 (new data points from 2 additional WT mice). This especially addressed some uneven datasets in figures 1-2 and 2, as pointed out by reviewer 1. We also performed new analysis of the electron microscopy images to deepen the understanding of morphological differences between genotypes and conditions. For this, we analyzed the active zone area of single active zones in boutons from WT and SynTKO animals, in treated and untreated conditions (Figure 5). We also analyzed the number of vesicles in vicinity to the active zone membrane (correlate for the readily-releasable pool) as well as the overall distribution of vesicle distances to the membrane (Figure 4-1). Furthermore, we improved the data visualization in figures 1, 1-2, 2, 4 and 5, as suggested by the reviewers. Our efforts resulted in the substantially revised version of our manuscript, that we are happy to submit today with the updated title: "The lack of synaptic alters presynaptic plasticity at hippocampal mossy fibers in male mice". The changes to the text, mainly in the results section and in the discussion, are marked in blue.

Below we enclose a point-by-point response to the reviewers' concerns.

Synthesis of Reviews:

Both Reviewers see potential value in the study, but they both raise serious concerns regarding different elements of the MS. Please address all of these concerns fully before resubmitting a substantially revised version. ___ Reviewer 1:

This is an interesting study, which reports new observations on functional and ultrastructural phenotypes in Syn TKO mossy fiber boutons. However, several technical and conceptual issues need to be addressed. Although electrophysiology data is solid, the EM part needs clarification and possibly additional work. Also, some aspects in the interpretation of Syn III function need to be clarified.

Major points:

1. Fig. 4. The panel A suggests that there may be problems with TEM quality: the vesicle do not seem clearly visibly, until they are outlined by the detection software. This raises the question of whether the software outlines and detects the vesicles reliably. Showing high magnifications micrographs with clearly seen vesicles could resolve this issue.

We thank Reviewer 1 for the careful revision of our manuscript and for the suggestion to improve the visualization of our data. We improved panel a) in figure 4, which now includes high magnification micrographs from WT and SynTKO synapses with clearly visible synaptic vesicles, without the detection software outlines (two images at the bottom of the panel). We also included two high magnification images from WT and SynTKO mossy fibers showing vesicles and active zones in the new supplementary figure 4-1. The striking reduction in vesicle density is clearly noticeable in the images from the SynTKO mossy fiber boutons.

2. Fig. 4 reports the reduced vesicle density in TKO. This was reported for every synapse studied for SynI/SynII DKO and also for SynII KO, and therefore this result is expected. The Discussion make implications about RRP and its relations to different forms of plasticity, however the authors do not try to assess RRP by EM. The EM results would have more merit if the authors quantify vesicles in the vicinity of AZs and make implications about RRP from their EM analysis.

We thank Reviewer 1 for raising this interesting point. We analyzed the number of vesicles and their distance to specific active zones. We plotted the number and proportion of vesicles within 40 nm from the active zone (bona fide correlate of the readily-releasable pool) to gain more insights into the general vesicle distribution as well as possible changes in the RRP.

Our data confirm once again the expected reduced overall vesicle density in the whole synapse (figure 4-1). Interestingly, this analysis further reveals a reduction in the absolute vesicle number within 40 nm from the active zone in boutons from SynTKO animals. When chemically potentiated with forskolin, though, both genotypes showed an increase in the vesicle number within 40 nm. These findings could indicate that parallel synapsin-independent mechanisms are in place that modulate the RRP during potentiation.

We did not include this analysis in our previous version of the manuscript because we are aware of technical limitations in the chemical fixation methodology as well as in the detection algorithm (we discussed these limitations in the updated method section and discussion). For these reasons, we now present the additional findings not in a main figure, but in supplemental figure 4-1. We believe that, while the high-pressure-freezing cryo-fixation method would have been a better choice, the analysis we present here gives us valuable insights for understanding the SynTKO phenotype. The changes we observed, are in line with previous work done with cryo fixation (Orlando et al., 2021; Kim et al., 2023). We revised the manuscript methods, results and discussion section to incorporate these additional findings.

3. Fig. 5. This figure needs to show unedited micrographs with identifiable AZs. It also needs clear description of AZ hallmarks and the evidence that they can be seen in unedited micrographs. As is, the figure only shows the results of the image analysis, and this is not acceptable.

We thank the reviewer for pointing out the need to clearly show active zones in raw images. We now improved figure 5 by adding unedited EM images from both genotypes and conditions (Figure 5a). There, active zones are indicated by black arrows. Briefly, we identified active zones as the area of membrane opposite to a postsynaptic density/spine profile and with vesicles attached and accumulated next to the membrane.

4. More detail is needed on how the mossy fiber boutons were identified. The methods section states: "The stratum lucidum of the hippocampal region CA3 was easily distinguishable for the presence of big mossy fiber boutons and for its localization just above the pyramidal cell layer." What magnification was used to determine the localization? How heterogeneous is the stratum lucidum? What criteria were used for the identification of mossy fiber boutons? This needs to addressed in detail and preferably illustrated.

We added a more detailed description of how we identified the mossy fiber boutons within the stratum lucidum to the method section. It reads like this: "A magnification of 7K x was used to determine the localization of mossy fiber boutons. Subsequently, synapses were imaged at 20 kx using a EM 900 Transmission Electron Microscope (Carl Zeiss, RRID:SCR_021364) operated at 80 keV and equipped with a Proscan 2K Slow-Scan CCD-Camera (Carl Zeiss). The stratum lucidum of the hippocampal region CA3 was easily distinguishable for the presence of big mossy fiber boutons and for its localization just above the pyramidal cell layer. Mossy fiber boutons were recognized by their large size, their position close to CA3 pyramidal cell bodies and a large number of presynaptic vesicles (Rollenhagen et al., 2007), including large clear and large dense-core vesicles." 5. The authors make parallels between DKO and TKO phenotypes in PTP and LTP and conclude that Syn III makes a major contribution here. What is known about the phenotype of single SynIII KO in PTP and LTP and how doe this agree with the conclusion of this manuscript? Cultured autaptic hippocampal neurons from SynIII KO animals have been investigated with electrophysiology (Feng et al. 2002). It was shown that while the transmission was normal in excitatory cells, inhibitory autapses from SynIII KO had significantly smaller currents. Paired-pulse facilitation was unchanged in excitatory neurons, but synaptic depression in response to 20 Hz trains was substantially lower in SynIII KO neurons.

In another study, cultured neurons from SynTKO showed reduced augmentation and potentiation in miniature excitatory postsynaptic currents when compared to WT. This phenotype could only be rescued via the SynIIIa isoform and was mediated via the cAMP-PKA pathway (Cheng et al. 2018). However, to our knowledge there is no study investigating the SynIII KO in regard to PTP or LTP.

PTP and LTP at the mossy fiber synapse have been investigated in SynDKO and SynI KO animals, but not in SynTKO. While PTP was suggested to be lower in mossy fibers of SynDKO animals, LTP was unchanged in these animals (Spillane et al. 1995). PTP and LTP were also described to be unchanged in SynI KO animals (Takei et al. 1995).

In light of our SynTKO characterization we conclude that it is likely that all synapsins participate in PTP, hence we see more severe impairment in this form of plasticity in SynTKO compared to findings from SynDKO. The fact that LTP is not altered in mossy fibers from SynDKO animals, instead, suggests that the phenotype that we describe might be due to the additional deletion of SynIII. This hypothesis is also supported by evidence that, in the absence of SynIII, axonal growth cones are structurally reorganized similarly to the mossy fibers. Also the proposed role for SynIII in the negative regulation of the recycling pool of vesicles supports our finding that the RRP seems reduced (Fig 4-1) (Piccini et al., 2015; Feng et al. 2002).

6. The datasets in figs 1-2 and 2 are very uneven: there are considerably fewer data points for WT We now performed additional recordings from 2 male WT mice, leading to more balanced data sets between SynTKO and WT in figure 1-2 and figure 2, with 11 recordings from 5 WT and 12 recordings from 6 SynTKO animals. To improve transparency, we additionally added a sentence to the study design section in the methods, which reads: "Due to the pooled data from the two presymptomatic SynTKO cohorts, there is an imbalance between numbers from WT and numbers from SynTKO animals in high-frequency recordings".

Minor:

Fig 1-2 Panels (b) and (c) have the same Y scale, and the scale in the panel (a) is different. Why? We adjusted the y-axis in Figure 1-2 panel a) to make the comparison between panels easier.

Fig. 2 It would be worthwhile to add WT versus KTO comparison at intermediate points, for example around 10th and 20th stimulus.

We added a comparison at 25th stimulus for the first and fourth stimulation train in figure 2 c) and d). We also added the description of those values and the statistics in table 7.

Reviewer 2:

The study by Bruentgens et al. addresses the physiological function of synapsin 3 at the mossy fiber/CA3 synapse of the mouse hippocampus. This is an important question because this synapse is one of the few places where synapsin 3 is expressed in the adult brain. The authors used electrophysiological recordings in hippocampal slices to compare synaptic transmission at the mossy fiber (MF) synapse in wild-type (WT) and synapsin triple knock out (TKO) mice. These measurements were complemented by EM analysis of the morphology of MF presynaptic terminals. Because synapsin TKO mice have seizures, the authors were commendably careful to compare WT and TKO mice at 2 different ages, before and after the onset of seizure activity.

The results described in the paper show that deletion of all synapsins increases fEPSP amplitude, impairs synaptic facilitation and early post tetanic potentiation (PTP). In addition, there is an increase in late PTP and LTP. Structurally, at MF synapses there is a large loss of synaptic vesicles - as previously reported for many other synapsin-deficient synapses - as well as an increased density of presynaptic active zones.

While I acknowledge the efforts of the authors, at the end of the day we still do not really know what synapsin 3 is doing at the mossy fiber synapse because of two fundamental issues with the paper, one technical and one more conceptual:

1. The technical issue is evident in the enhancement of fEPSP amplitude shown in Fig. 1b (and 1.1b). This would indicate a huge increase in glutamate release from the MFs in TKO mice, a result that is at odds with decades of research on the effects of synapsins on glutamate release (as well as the absence of effects on synaptic facilitation, as shown in Fig. 1.2 of the paper). In lines 574-584 of the text, the authors themselves point out that this paradoxical result is likely due to the (unmeasured) contributions of inhibitory synapses, which in fact are preferentially affected by loss of synapsins. Given this confound, it becomes almost impossible to interpret the results shown in Figures 1-3 because the authors are measuring some ill-defined mixture of effects on excitatory and inhibitory synaptic transmission. The fact that DCG-IV blocks at least 75% of the synaptic response (e.g. Figure 3a,c) is not able to solve this problem, because of the possible feedback inhibition between mossy fibers/CA3 neurons and interneurons. I really do not understand why the authors did not sidestep this serious problem by performing their measurements in the presence of GABA receptor blockers.

We thank reviewer 2 for pointing out this technical issue.

For this study we did not perform experiments with GABA receptors blockers. The reasoning behind this decision was that GABA receptor blockers in the excitable SynTKO slices would, in our experience, induce epileptic activity in the slice. A possible solution to this problem could have been the addition of both GABA receptor blockers and AMPA receptor blockers to prevent epileptic activity and to subsequently measure NMDAR currents. We did not perform these experiments as they were beyond the scope of our current investigation.

We would like to clarify that we did not claim that the input/output curve is due to a massive increase in glutamate release. We see similar increases in excitability as Farisello and coworkers did see in area CA1 and we discussed the contribution of inhibition as a major mechanism for excitability and epileptogenesis (see also Farisello et al., 2013). Furthermore, an increase in glutamate release would not be the only option leading to the effects we see. A change in pre- and/or postsynaptic receptor composition could for example be involved and it has indeed been described that glutamate receptor levels increased in area CA3 of SynDKO mice (Owe et al. 2005). In fact, with our new finding of reduced RRP size in SynTKO animals we rather see a structural correlate for a decrease in release probability. This finding also fits to our observed trend in the increase of PPR (Figure 1-2), because a smaller RRP is associated with a lower release probability, as is an increased PPR. We tried to be careful to not over interpret those tendencies, because with field recordings this measure can be confounded.

2. Beyond this important technical shortcoming, the conceptual issue is that the results in the paper do not allow us to make any real conclusions about the function of synapsin 3 because the mossy fibers of TKO mice have lost all synapsins (1,2 and 3 genes), rather than synapsin 3 alone. The authors attempt to work around this problem by comparing their results to those published previously on synapsin double knockout (or single knockout) mice (e.g. Spillane et al., 1995; Owe et al., 2009). While such comparisons seem reasonable at first, in fact the experimental conditions differ substantially between the results reported in each paper. To give just one example, the protocol used by the authors to evoke activity-dependent facilitation of synaptic transmission (0.05 Hz stimulation followed by 1 Hz stimulation for 20 min) is rather different from the protocol used by Owe et al. (2009): 0.1 Hz stimulation followed by 2 Hz stimulation for 15 min. Different patterns of activity surely will elicit differing amounts of plasticity and may even engage different plasticity mechanisms, making a direct comparison difficult. Similar arguments apply to the striking change in LTP observed in TKO slices (Figure 3c). To yield definitive conclusions, the authors should perform side-by-side comparisons between different genotypes in identical experimental conditions.

We thank the reviewer 2 for his or her suggestions. We agree that the interpretation of the SynTKO results is confounded by the deletion of SynI and SynII. As we mention in the discussion, in this exploratory study we wanted to exclude possible compensatory effects due to the presence of SynI and II in SynIII single KO animals. With this work we gained knowledge regarding frequency facilitation, PTP and LTP in the absence of all synapsins. We agree that future work aimed at characterising SynIII KO mossy fiber structure and function will be important to specifically investigate SynIII role in adult mossy fiber synapses. We now removed all conclusions specifically regarding SynIII and, in general, toned down our claims.

Reviewer 2 points also out, that we use different protocols compared to Owe et al. (2009) as well as Spillane et al. (1995). We want to elaborate on our choice of protocols and thereby underline the robustness of our approach.

Owe and co-workers used a 2 Hz stimulation paradigm while we made use of a 1 Hz stimulation paradigm. We believe that both are suited to evoke mossy fiber facilitation due to the unique physiology of this synapse. These synapses are able to facilitate in a wide range of frequencies, described by Salin and others (Salin et al., 1996; Nicoll and Schmitz, 2005). At frequencies as low as 0.33 Hz, they can facilitate 5- fold (Salin et al., 1996, Figure 4), comparable to the levels we observe in WT animals at 1 Hz (6.5 fold) and Owe et al. at 2 Hz (4 fold). Owe et al. used a baseline frequency of 0.1 Hz and increased it to 2 Hz as the facilitating stimuli (Owe et al., 2009). The ratio between our baseline and stimulation frequency was the same, with a baseline frequency of 0.05 Hz and a stimulation frequency of 1 Hz. Although Owe et al. stimulated substantially longer at 2 Hz than we did at 1 Hz, they show a zoom-in to the first three minutes of their recording, making it feasible to compare their initial facilitation levels within a similar time frame to ours.

Spillane and coworkers used an induction protocol with 100 Hz trains to induce PTP and LTP (Spillane et al., 1995), while we decided to use a 25 Hz protocol in our study. This latter protocol is used for decades as an alternative to induce mossy fiber LTP and is comparable to a 100 Hz induction protocol (Mellor and Nicoll, 2001).

In addition to these fundamental problems, here are a few minor issues for the authors to consider:

1. In the long-stimulation experiments (Figures 2a and b), the amplitude of the first fEPSP in synTKO is smaller than that of WT, which is inconsistent with the effect shown in Figure 1. The authors need to explain this discrepancy.

We agree with reviewer 2 that it seems contradictory that the example traces in Figure 2a seem to have smaller fEPSP amplitudes in the SynTKO, although the example traces in Figure 1b show the opposite. However, this is the result of a necessary recording paradigm: the choice of stimulation strength. In our input-output recordings we varied the stimulation strength and saw a steeper increase in the relation between PFV and fEPSP for SynTKO than for WT animals when increasing the stimulation strength (Figure 1b). However, most data points from SynTKO are situated in the lower left corner, indicating a small fEPSP amplitude. To avoid epileptiform activity during our slice recordings and to allow for activity-dependent increase in the responses, we chose a non-saturating stimulation strength for the recordings after the input/output curve. In figure 1b our main goal was to show that with a similar number of stimulated fibers (measured via the PFV), the fEPSP amplitude is larger for SynTKO animals. To make it clearer, we now marked the data points referring to the example traces in Figure 1b with a black arrow.

To further explain the apparent conundrum, we compared averaged absolute amplitudes from the baseline before the LTP recording, resulting from the moderate, non-saturating stimulation strength. We found that the average baseline fEPSP amplitude was more variable in SynTKO animals than in WT, with a coefficient of variation of 61.3% for SynTKO and 30.84% for WT. We also found a tendency for the median baseline amplitude to be smaller in SynTKO than in WT animals with a median amplitude of 0.202 [0.136; 0.241] mV in WT and of 0.16 [0.09; 0.21] mV for SynTKO (p = 0.065, Mann Whitney U test). However, when actually measuring the first fEPSP amplitudes from the given two example traces in Figure 2a, we find comparable amplitudes with 0.121 mV for the WT trace and 0.118 mV for the SynTKO trace. We realized that our example traces in Figure 2 might have been a bit too small to detect such details, so we increased their size. Regarding Figure 2b we want to mention that due to the normalization of the fEPSPs the first amplitudes of both genotypes almost co-localize, making it hard to spot them.

2. The authors showed an interesting increase in active zone density in TKO mice compared to WT. However, because the total area of the AZ is also a factor that affects glutamate release, the authors should measure AZ area.

We thank the reviewer for pointing out this aspect. We measured the AZ area in both conditions and found it to be unchanged, in line with results from previous work in control versus potentiated WT boutons (Orlando et al., 2021). We now added the results of this analysis to figure 5 panel d) as well as to the text: "the forskolin-induced increase in docked and tethered vesicles was not accompanied by a change in the active zone area of mossy fiber boutons (Orlando et al., 2021). To test if this holds true in SynTKO animals, we analysed the area of individual active zones from partial 3D reconstructions of treated and untreated WT and SynTKO boutons. Mean active zone areas per bouton were comparable for both genotypes and treatments (Figure 5d). We fitted a generalized linear mixed model (Gamma family with log link) to estimate active zone area given the genotype and treatment, including the individual boutons as random effects. When comparing the model to nested null models, we found no evidence for an effect of treatment (p = 0.5846), genotype (p = 0.593) or the interaction of both (p = 0.8113) on the active zone areas. Thus, the active zone density increases while the active zone area is unchanged." 3. One (very minor) annoyance is that the authors italicize boutons throughout the text. This definitely is not the usual practice, because boutons is now considered to be a standard English word, despite its French origins! We now changed all "boutons" to regular font.
