# Erratum: Bruentgens et al., “The Lack of Synapsin Alters Presynaptic Plasticity at Hippocampal Mossy Fibers in Male Mice”

**DOI:** 10.1523/ENEURO.0360-24.2024

**Published:** 2024-09-10

In the article “The Lack of Synapsin Alters Presynaptic Plasticity at Hippocampal Mossy Fibers in Male Mice” by Felicitas Bruentgens, Laura Moreno Velasquez, Alexander Stumpf, Daniel Parthier, Jörg Breustedt, Fabio Benfenati, Dragomir Milovanovic, Dietmar Schmitz, and Marta Orlando which was published online on June 12, 2024, the arrow in the third row and third column of Table 8 should indicate downward rather than upward to accurately represent the depression at SynIII knockouts. This refers to findings (Feng et al. 2002), where depression was found to be defective in SynIII knockouts compared with wild-type synapses. The authors apologize for the error. The changes do not affect the conclusions of the paper, and the online version has been corrected.

**Table 8.** Overview of functional and structural changes in glutamatergic synapses of synapsin KOs

Genotype	Syn I KO	Syn II KO	Syn III KO	Syn DKO	SynTKO	References
Excitability		↑			↑(**↑**)	Boido et al. (2010); Farisello et al. (2013); Matos et al. (2019) (**this work**)
Frequency facilitation	↓	=		↓	(**↓**)	**Owe et al. (2009)**; Nikolaev and Heggelund (2015) (**this work**)
Depression	=↑	=↑	↓	↑	↑(**↑**)	Rosahl et al. (1995); Feng et al. (2002); Gitler et al. (2004); Sun et al. (2006); Vasileva et al. (2012); Farisello et al. (2013) (**this work**)
PTP	=↓	=↓		↓	↓(**↓**)	Rosahl et al. (1993, 1995); **Spillane et al. (1995)**; Kielland et al. (2006); Valente et al. (2012); Farisello et al. (2013); Nikolaev and Heggelund (2015); Cheng et al. (2018) (**this work**)
LTP	**=**			**=**	(**↑**)	Rosahl et al. (1993, 1995); **Spillane et al. (1995)**; **Takei et al. (1995)** (**this work**)
Density of synaptic vesicles	**↓**		**=**	**↓**	↓(**↓**)	Li et al. (1995); Pieribone et al. (1995); Rosahl et al. (1995); **Takei et al. (1995)**; **Feng et al. (2002)**; Gitler et al. (2004); Siksou et al. (2007); **Owe et al. (2009)**; Vasileva et al. (2012) (**this work**)

The arrows indicate an increase or decrease of the properties listed in the first column, while the equals signs indicate no changes. Bold symbols indicate that previous work also considered the hippocampal mossy fiber bouton. References are marked accordingly. The arrows in brackets refer to the findings of this study.
